# Brainstem neuromelanin and iron MRI reveals a precise signature for idiopathic and LRRK2 Parkinson’s disease

**DOI:** 10.1038/s41531-023-00503-2

**Published:** 2023-04-15

**Authors:** Martín Martínez, Mikel Ariz, Ignacio Alvarez, Gabriel Castellanos, Miquel Aguilar, Jorge Hernández-Vara, Núria Caballol, Alicia Garrido, Àngels Bayés, Dolores Vilas, Maria Jose Marti, the Catalonian Neuroimaging Parkinson’s disease Consortium, Berta Pascual-Sedano, Juan Marin, Asuncion Avila, Mariateresa Buongiorno, Juan Pablo Tartari, Victor Puente, Mario Ezquerra, Francesc Valldeoriola, Yaroslau Compta, Eduard Tolosa, Claustre Pont, Pau Pastor, Carlos Ortiz de Solórzano, Maria A. Pastor

**Affiliations:** 1 Neuroimaging Laboratory, University of Navarra, School of Medicine, Pamplona, Spain; 2 School of Education and Psychology, University of Navarra, Pamplona, Spain; 3 Ciberonc and Solid Tumours and Biomarkers Program, CIMA University of Navarra, Pamplona, Spain; 4 Movement Disorders Unit, Neurology, University Hospital Mútua de Terrassa, Terrassa, Barcelona, Spain; 5 Department of Physiological Sciences, Facultad de Medicina, Pontificia Universidad Javeriana, Bogotá, Colombia; 6 Neurology Department, Hospital Universitari Vall D´Hebron, Neurodegenerative Diseases Research Group, Vall D’Hebron Research Institute (VHIR), Barcelona, Spain; 7 Department of Neurology, Complex Hospitalari Moisès Broggi, Sant Joan Despí, Barcelona, Spain; 8 Parkinson and Movement disorders Unit, Hospital Quirón-Teknon, Barcelona, Spain; 9 Parkinson’s Disease and Movement Disorders Unit, Neurology Service, IDIBAPS, CIBERNED, Centro de Investigación Biomédica en Red Sobre Enfermedades Neurodegenerativas: CB06/05/0018-ISCIII), ERN-RND Hospital Clínic i Provincial de Barcelona, Barcelona, Catalonia Spain; 10 Department of Medicine & Institut de Neurociències of the University of Barcelona, Barcelona, Catalonia Spain; 11 Unit of Neurodegenerative diseases, Department of Neurology, University Hospital Germans Trias i Pujol, Badalona, Spain; 12 Neurosciences, The Germans Trias i Pujol Research Institute (IGTP) Badalona, Badalona, Catalonia Spain; 13 Neurosciences, School of Medicine, University of Navarra, Pamplona, Spain; 14 Movement Disorders Unit, Neurology, Hospital Santa Creu i Sant Pau, Universitat Autònoma de Barcelona, Barcelona, Spain; 15 Movement Disorders Unit, Neurology, Hospital del Mar, Barcelona, Catalonia Spain; 16 Neurology Service, Hospital General de Granollers, Granollers, Spain

**Keywords:** Diagnostic markers, Parkinson's disease

## Abstract

Neuromelanin (NM) loss in substantia nigra pars compacta (SNc) and locus coeruleus (LC) reflects neuronal death in Parkinson’s disease (PD). Since genetically-determined PD shows varied clinical expressivity, we wanted to accurately quantify and locate brainstem NM and iron, to discover whether specific MRI patterns are linked to Leucine-rich repeat kinase 2 G2019S PD (LRRK2-PD) or idiopathic Parkinson’s disease (iPD). A 3D automated MRI atlas-based segmentation pipeline (3D-ABSP) for NM/iron-sensitive MRI images topographically characterized the SNc, LC, and red nucleus (RN) neuronal loss and calculated NM/iron contrast ratio (CR) and normalized volume (nVol). Left-side NM nVol was larger in all groups. PD had lower NM CR and nVol in ventral-caudal SNc, whereas iron increased in lateral, medial-rostral, and caudal SNc. The SNc NM CR reduction was associated with psychiatric symptoms. LC CR and nVol discriminated better among subgroups: LRRK2-PD had similar LC NM CR and nVol as that of controls, and larger LC NM nVol and RN iron CR than iPD. PD showed higher iron SNc nVol than controls, especially among LRRK2-PD. ROC analyses showed an AUC > 0.92 for most pairwise subgroup comparisons, with SNc NM being the best discriminator between HC and PD. NM measures maintained their discriminator power considering the subgroup of PD patients with less than 5 years of disease duration. The SNc iron CR and nVol increase was associated with longer disease duration in PD patients. The 3D-ABSP sensitively identified NM and iron MRI patterns strongly correlated with phenotypic PD features.

## Introduction

Neuromelanin (NM), located mainly in the *substantia nigra pars compacta* (SNc) dopaminergic neurons and in the noradrenergic neurons at the *locus coeruleus* (LC)^1^, is involved in long-term neuronal iron immobilization and oxidative processes^2^. In the early stages of Parkinson’s disease (PD), SNc dopaminergic neurons show Lewy bodies composed of α-synuclein, which mobilizes NM into granules^3^. By the time of diagnosis, 30–50% of dopaminergic neurons in the SNc are lost, and NM loss follows a mono-exponential decline corresponding with disease progression, occurring faster in early stages^4,5^.

Early studies of NM-sensitive magnetic resonance imaging (MRI) sequences (NM-MRI) applied to pathological specimens revealed a correlation between NM reduction and SNc and LC neuronal loss^6,7^, thus NM-MRI can become a diagnostic tool to distinguish PD patients from healthy controls (HC)^7–9^ and can help quantify NM loss and delimitate severely affected regions within SNc and LC. The decrease of NM-containing dopaminergic neurons inversely correlates with iron content as the disease progresses, suggesting that iron overload facilitates neuronal death^10^. Therefore, further research on the connecting links between NM loss and iron overload in SNc and LC could provide imaging biomarkers of PD progression^11^. Furthermore, the role of the red nucleus (RN) in PD has not been fully explained. RN is an iron-enriched structure, which along with the neighboring *substantia nigra* plays a role in motor coordination, compensating the dysfunctional thalamo-striatal-cortical circuit in PD^12^,

*Leucine-rich repeat kinase 2* (*LRRK2;* PARK8; MIM:609007) G2019S mutation is responsible for ~5% of familial and ~1% of sporadic PD^13,14^. Since recent therapies are targeting LRRK2 kinase activity, we found relevant to study LRRK2-PD different regional vulnerability of brainstem dopaminergic neurons to achieve specific neuroimaging signatures that can help to easily monitor disease staging.

**Fig. 1:**
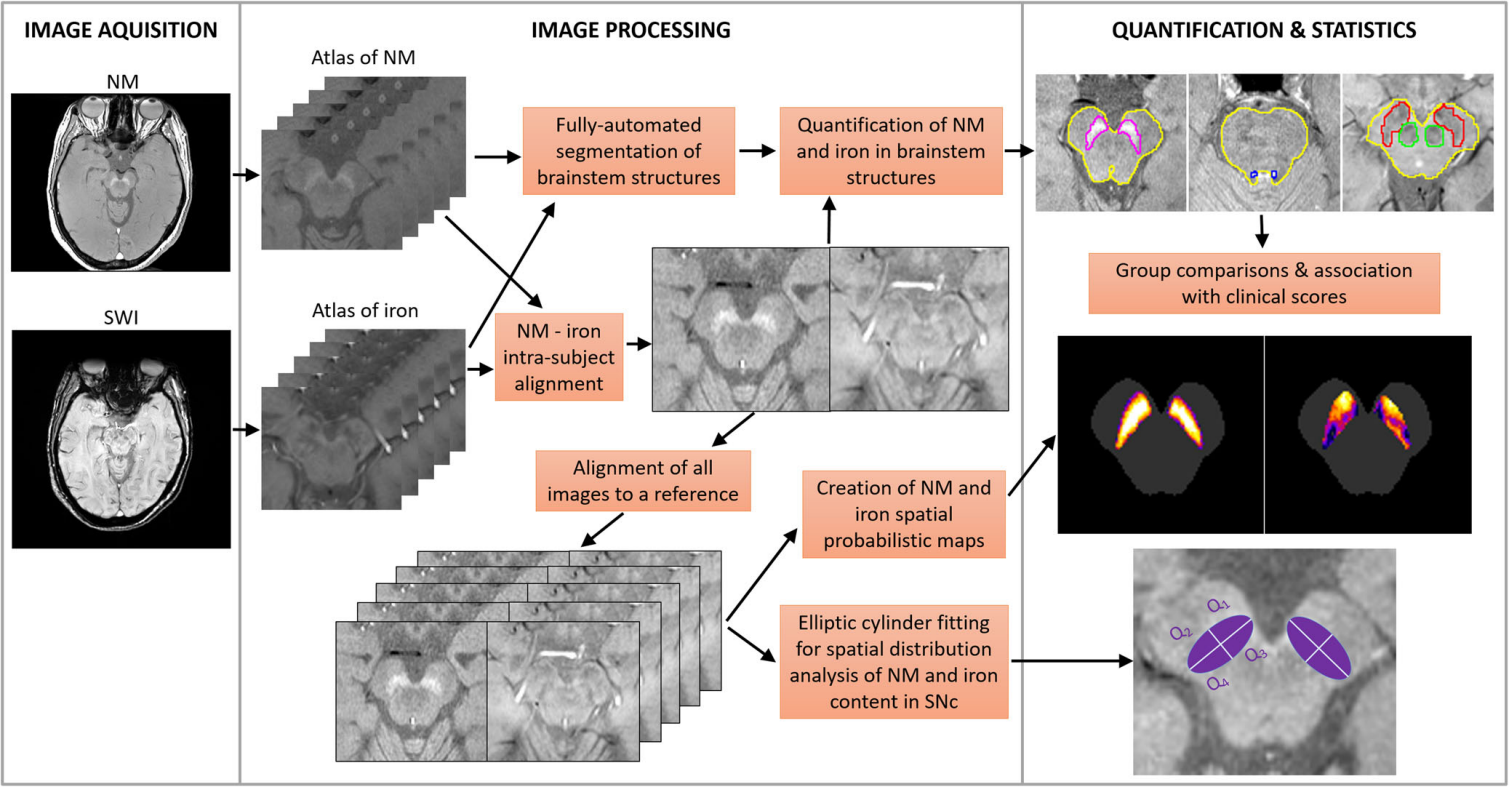
Preprocessing, segmentation and quantification of MRI NM and Iron images. 3D automated atlas-based segmentation pipeline (3D-ABSP) workflow diagram for NM and iron quantification in the brainstem.

The aim of this study was to perform an accurate quantification/topography of NM in the SNc and LC and iron in the SNc and RN, using an automated brainstem atlas-based segmentation pipeline (3D-ABSP) optimized from previous work^15^, in order to elucidate the NM and iron content patterns in *LRRK2-*PD and iPD. 3D-ABSP allows the accurate alignment of NM and iron images, colocalization of NM and iron in the SNc, LC, and RN, and unbiased quantification of the contrast ratio (CR) and normalized volume (nVol) of NM and iron in those structures (Fig. 1). We hypothesized that, by using 3D-ABSP, we could discover specific neuroimaging signatures in both idiopathic and genetic PD, for which an accurate early diagnosis could lead to new specific treatments.

## Results

### Subjects’ recruitment and genetic analysis

Sixty-three PD patients joined the study between April 2018 and October 2020, along with thirty-two HC, matched for age and disease duration. Ninety-one subjects (96%) were right-handed and four (4%) were ambidextrous, according to the *Edinburgh Handedness Inventory*^16^. Twenty-four patients were stratified as G2019S *LRRK2* mutation carriers (LRRK2-PD) and thirty-nine as idiopathic PD subjects without the G2019S *LRRK2 or GBA* mutations (iPD). There were no statistically significant differences in age, sex, years of education, disease duration, non-motor or cognitive variables among PD subgroups, although Unified Parkinson’s Disease Rating Scale part III (UPDRS-III) and Parkinson’s Disease Cognitive Rating Scale (PD-CRS) scores differed slightly between iPD and LRRK2. Rapid eye movement (REM) sleep behavior disorder (RBD) was more prevalent in iPD (*p* = 0.01) than in LRRK2-PD (Table 1).

**Table 1:** Demographic and clinical data of the cohort.

	HC (*n* = 32)	iPD (*n* = 39)	*LRRK2* G2019S carriers (*n* = 24)	Group comparisons
Age	63.53 ± 8.21 [51–81]	65.15 ± 9.97 [39–80]	66.04 (10.87) [43–83]	*F* = 1.40, *p* = 0.26
Sex (F/M)	17 (53.1%)/15 (46.9%)	17 (43.6%)/22 (56.4%)	14 (58.3%)/10 (41.7%)	*χ*² = 1.41, *p* = 0.50
Handedness (R/A)	30 (93.7%)/2 (6.3%)	38 (97.4%)/1 (2.6%)	23 (95.8%)/1 (4.2%)	*χ*² = 0.59, *p* = 0.75
More affected body side (L/R/B)	n.a.	18 (46.1%)/20 (51.3%)/1 (2.6%)	14 (58.3%)/9 (37.5%)/1 (4.2%)	*χ*² = 1.17, *p* = 0.72
Years of education	n.a.	10.15 ± 5.12 [0–19]	9.67 ± 4.89 [0–20]	*t* = 0.06, *p* = 0.95
Age at onset	n.a.	56.56 ± 11.51 [25–76]	57.08 ± 11.51 [40–68]	*F* = 0.06, *p* = 0.95
Disease duration	n.a.	8.59 ± 5.38 [2–23]	8.96 ± 5.39 [2–20]	*F* = 0.10, *p* = 0.92
H&Y stage	n.a.	2.04 ± 0.31 [2–2.5]	2.08 ± 0.84 [1–5]	*χ*² = 0.01, *p* = 0.99
UPDRS-III	n.a.	21.18 ± 8.18 [5–41]	16.67 ± 13.16 [2–45]	*F* = 2.20, *p* = 0.04
LEDD	n.a.	724.03 ± 383.81 [0–1610]	593.88 ± 384.54 [50–1570]	*F* = 1.45, *p* = 0.15
Psychiatric symptoms	n.a.	2 (5.1%)	4 (16.7%)	*χ*² = 2.30, *p* = 0.19
NMSS	n.a.	29.84 ± 24.11 [0–99]	20.86 ± 18.74 [3–77]	*F* = 1.41, *p* = 0.17
Hyposmia	n.a.	20 (51.3%)	7 (29.2%)	*χ*² = 2.97, *p* = 0.12
Other sleep disorders	n.a.	22 (56.4%)	16 (66.7%)	*χ*² = 0.65, *p* = 0.44
RBD	n.a.	24 (61%)	6 (25%)	*χ*² = 7.95, *p* = 0.01
MMSE	n.a.	26.62 ± 2.61 [22–30]	26.65 ± 2.64 [20–30]	*F* = 0.50, *p* = 0.62
PD-CRS	n.a.	87.41 ± 17.45 [55–131]	95.65 ± 18.34 [57–126]	*F* = 2.03, *p* = 0.05

Chi-Square and Kruskal-Wallis tests were applied to compare categorical variables between two and three groups, respectively; whereas robust *t tests* and heteroscedastic one-way ANOVAs were applied to compare quantitative continuous variables between two and three groups, respectively. All tests were corrected for multiple comparisons with Bonferroni’s method. Quantitative data are shown as mean, standard deviation (in parenthesis), and range values (in brackets), while categorical data are shown as frequency and percentages. *F* female, *M* male, *R* right, *L* left, *A* ambidextrous, *B* bilateral. *H&Y* Modified Hoehn & Yahr Stage, *UPDRS-III* Unified Parkinson’s Disease Rating Scale part III, *LEDD* levodopa equivalent daily dose, *NMSS* non-motor symptoms scale for Parkinson’s Disease, *RBD* rapid eye movement (REM) sleep behavior disorder, *MMSE* mini-mental state examination, *PD-CRS* Parkinson’s Disease Cognitive Rating scale, *n.a.* not applicable/available.

### Quantification of NM CR and nVol in SNc and LC

**Fig. 2:**
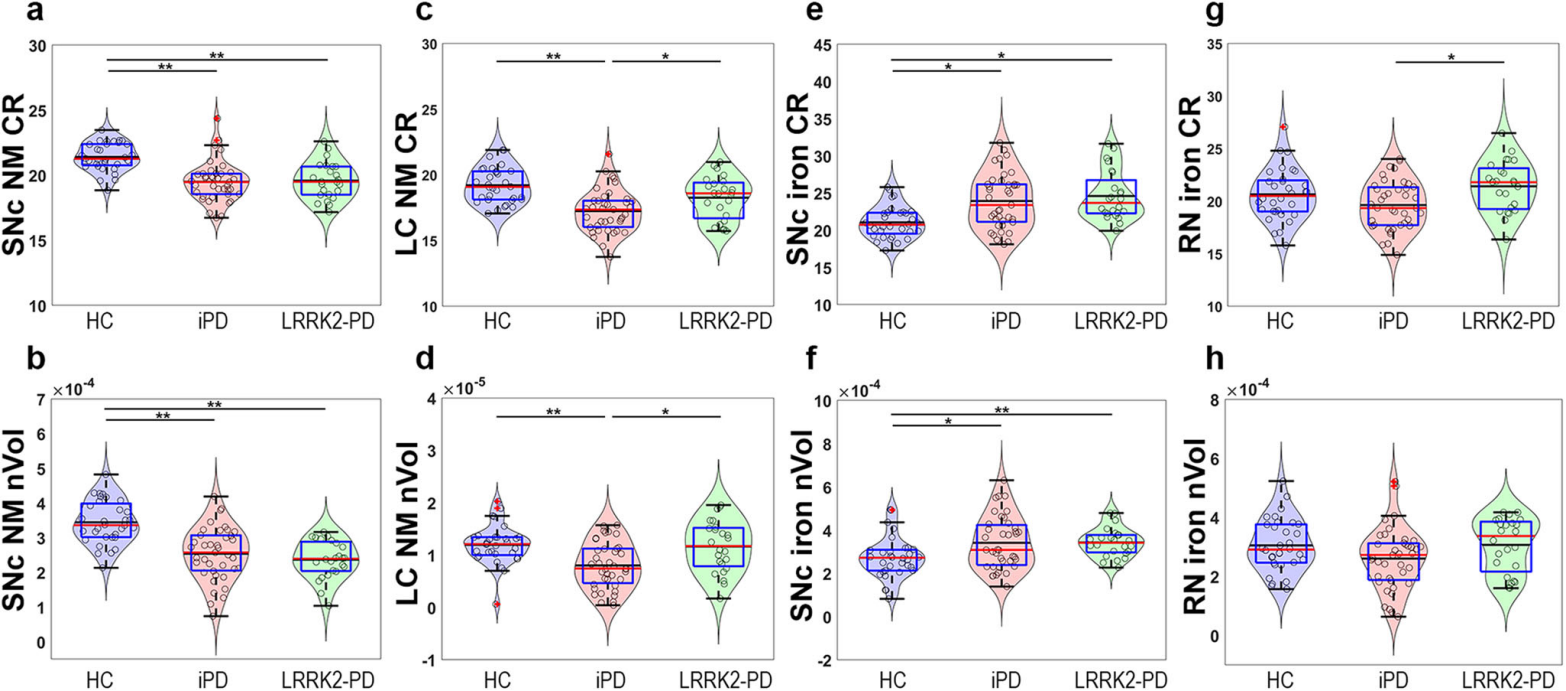
Box and violin plots of the quantitative brainstem MRI measures. Data is represented with a box plot (box edges: first 25th percentile quartile to third 75th percentile quartile; red line: median; black line: mean; whisker length: 1.5 times the interquartile range; red crosses: outlier data), a violin plot showing the histogram distribution, and individual scatter points of each subject’s quantitative measures. **a** SNc NM CR, **b** SNc NM nVol, **c** LC NM CR, **d** LC NM nVol, **e** SNc iron CR, **f** SNc iron nVol, **g** RN iron CR, and **h** RN iron nVol. Red lines represent the median and black lines represent the mean, whereas gray horizontal lines indicate statistically significant differences between groups (*p < 0.05, **p < 0.001, corrected for multiple testing).

Both SNc CR (Fig. 2a) and nVol (Fig. 2b) were higher in HC than iPD and LRRK2-PD groups. LC analysis showed robust differences among groups for the CR (Fig. 2c). *Post hoc* tests revealed that HC and LRRK2-PD had higher LC CR than iPD. Similarly, there were statistically significant LC nVol differences (Fig. 2d), since HC and LRRK2-PD had larger LC nVol than iPD. No differences in LC CR and LC nVol were found between LRRK2-PD and HC groups suggesting LC preservation among LRRK2-PD when the disease progressed (Supplementary Table 1).

### Quantification of iron CR and nVol in SNc and RN

SNc iron values substantially differed across groups: HC displayed smaller SNc iron nVol than iPD and LRRK2-PD (Fig. 2f) and lower SNc iron CR than iPD and LRRK2-PD (Fig. 2e).

RN iron CR showed differences between groups (Fig. 2g). Post hoc tests indicated higher RN iron CR in LRRK2-PD than iPD. No statistically significant RN iron nVol differences were found across groups (Fig. 2h).

NM and iron CR and nVol-specific patterns among PD subgroups in reference to HC in brainstem structures are reflected in Table 2. Information on automatic segmentation and between-sequence image alignment quality-control scores are detailed in Supplementary Results.

**Table 2:** Summary of brainstem NM and iron CR and nVol behavior among PD subgroups in reference to the HC.

Sequence	Region	Parameter	HC	iPD	LRRK2-PD
NM	SNc	CR	—		
		nVol	—		
	LC	CR	—		—
		nVol	—		—
Iron	SNc	CR	—		
		nVol	—		
	RN	CR	—	—	—
		nVol	—	—	—

Single black arrows indicate statistically significant differences (*p* < 0.05, *d* < 0.8), double black arrows indicate statistically significant differences with large effect size (*p* < 0.05, *d* ≥ 0.8), and white arrows indicate statistical trends.

### Quantification of iron in SNc vs. substantia nigra pars reticulata (SNr)

A two-way mixed ANOVA performed to assess group and region (SNc/SNr) effects of iron CR and nVol showed a main effect of region for the CR (*F* = 18.7, *p* < 0.001, SNr > SNc) and a group x region interaction (*F* = 12.41, *p* < 0.001). Specifically, HC had lower CR in the SNc than in the SNr. A main effect of region was found for the nVol (*F* = 277.38, *p* < 0.001) meaning that iron nVol was larger in SNr than in the SNc in all groups (Supplementary Fig. 1).

### Analysis of NM and iron side asymmetry and topographical interaction

In all subjects, a volumetric asymmetry (left > right) was consistently found for both NM and iron in the SNc and also in NM LC, but not in the RN iron (Supplementary Fig. 2, Supplementary Table 2). In the SNc, the asymmetry was mainly due to differences in Q_1_ (corresponding to the ventral SNc) and Q_2_ (corresponding mostly to the lateral SNc) quadrants (Supplementary Figs. 3 and 4).

**Fig. 3:**
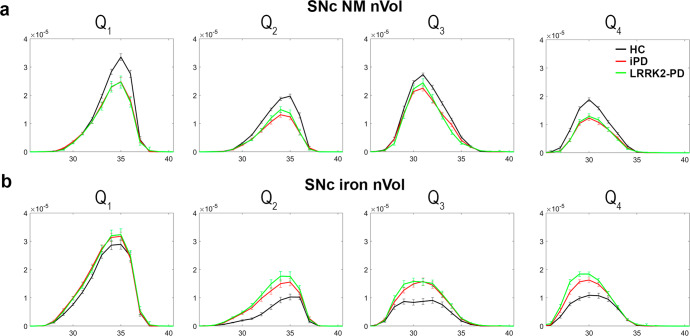
Topographical distribution of NM and iron nVol in the SNc. Distribution of **a** NM nVol; and **b** iron nVol in the SNc according to PD subtypes, anatomical quadrants, and slices, from caudal (Q1) to dorsal (Q4).

The two-way ANOVA on the effect of group and sequence (NM/iron) in the SNc revealed a decrease of both SNc NM CR and nVol along with an increase of both SNc iron CR and nVol (Supplementary Fig. 5 and Supplementary Table 3). The SNc NM nVol loss in PD was more pronounced in Q_1_ and Q_2_ quadrants, whereas the increased iron burden laid in Q_2_, Q_3_, and Q_4_ quadrants (Fig. 3 and Supplementary Figs. 3 and 4).

**Fig. 4:**
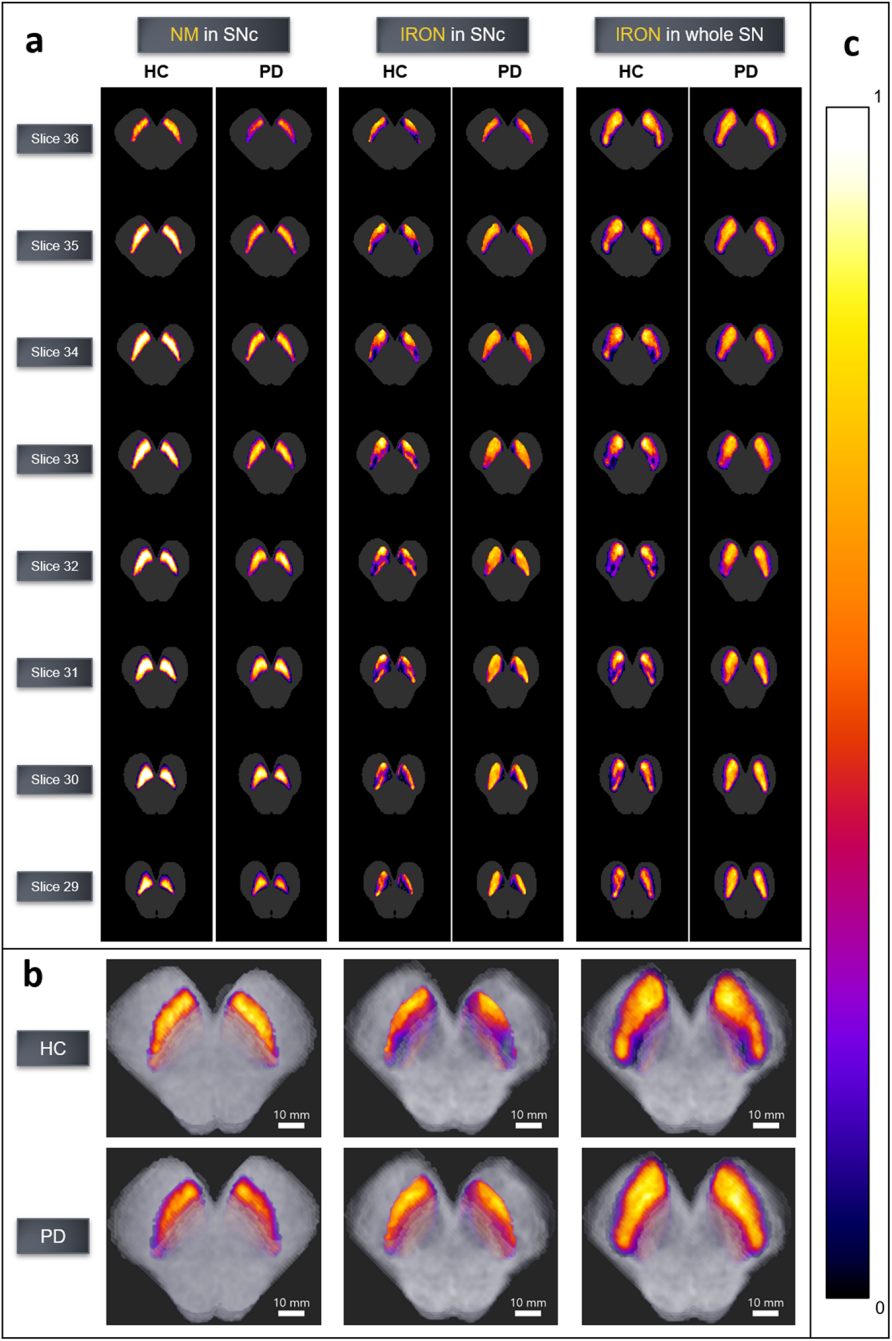
SNc NM and iron probabilistic maps. **a** 2D axial slices of probabilistic maps of NM and iron obtained from healthy subjects and PD patients. **b** 3D renders of HC and PD for NM in SNc (left), iron in SNc (middle), and iron burden in SN (right). **c** Colorbar for normalized voxel NM/iron content. Average brainstem mask is shown in gray. The probabilistic map shows the voxels for which most subjects contain NM/iron (warm colors) and the voxels for which few subjects contain NM/iron (cold colors). To improve visual quality, all maps were spatially upsampled by a factor of four using bicubic interpolation.

**Fig. 5:**
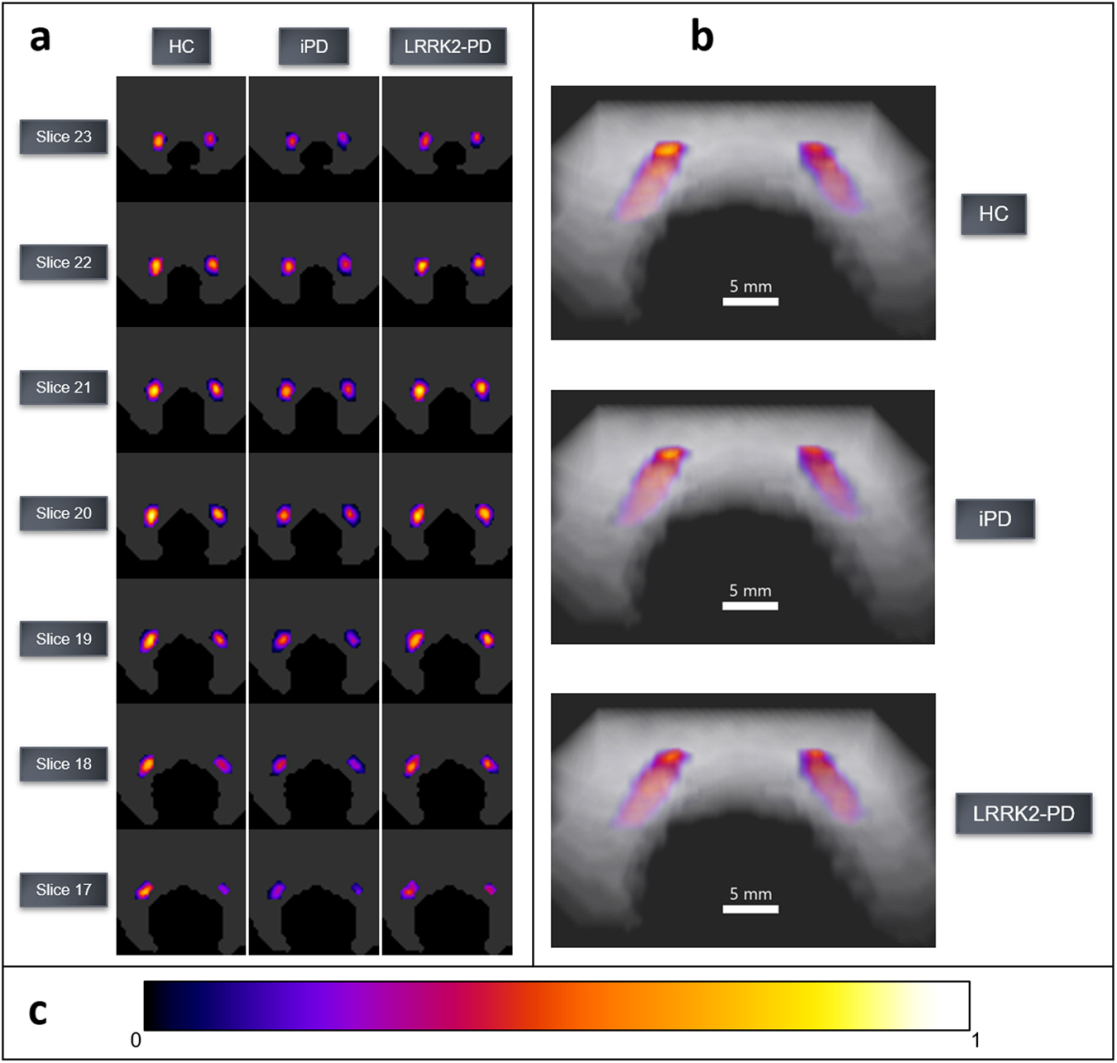
Probabilistic maps of the LC NM content in HC, iPD, and LRRK2-PD groups. **a** 2D-slice axial visualization of the probabilistic maps, **b** 3D volumetric renders, and **c** Colorbar for the normalized NM content per voxel. Average brainstem mask is shown in gray. The probabilistic map shows the voxels for which most subjects contain NM (warm colors) and the voxels for which few subjects contain NM (cold colors). To improve visual quality, all maps were spatially upsampled by a factor of four using bicubic interpolation.

SNc NM and iron probabilistic maps (Fig. 4) showed a reduced NM content as well as an iron increase in SNc in the PD group compared with HC. The iron map obtained from HC allowed us to accurately distinguish the Nigrosome-1 (NG-1) and 2 (NG-2). LC NM probabilistic maps (Fig. 5) displayed similar NM content in HC and LRRK2-PD groups, and reduced NM content in iPD (Table 1).

### ROC analyses for evaluation of diagnostic performance

To assess the diagnostic potential of the 3D-ABSP tool, eight MRI quantitative variables (SNc NM CR and nVol, SNc iron CR and nVol, LC NM CR and nVol, and RN iron CR and nVol; Fig. 2) were analyzed individually or combined through binary logistic regression. Since the LC is the structure that showed the most differences between PD subgroups, we first analyzed the ROC curves of LC NM CR and LC NM nVol. Secondly, both parameters were combined through logistic regression analysis. Thirdly, RN iron CR and nVol values were added to the model, followed by the addition of the SNc NM CR and nVol, and finally the SNc iron CR and nVol for the complete regression model.

**Fig. 6:**
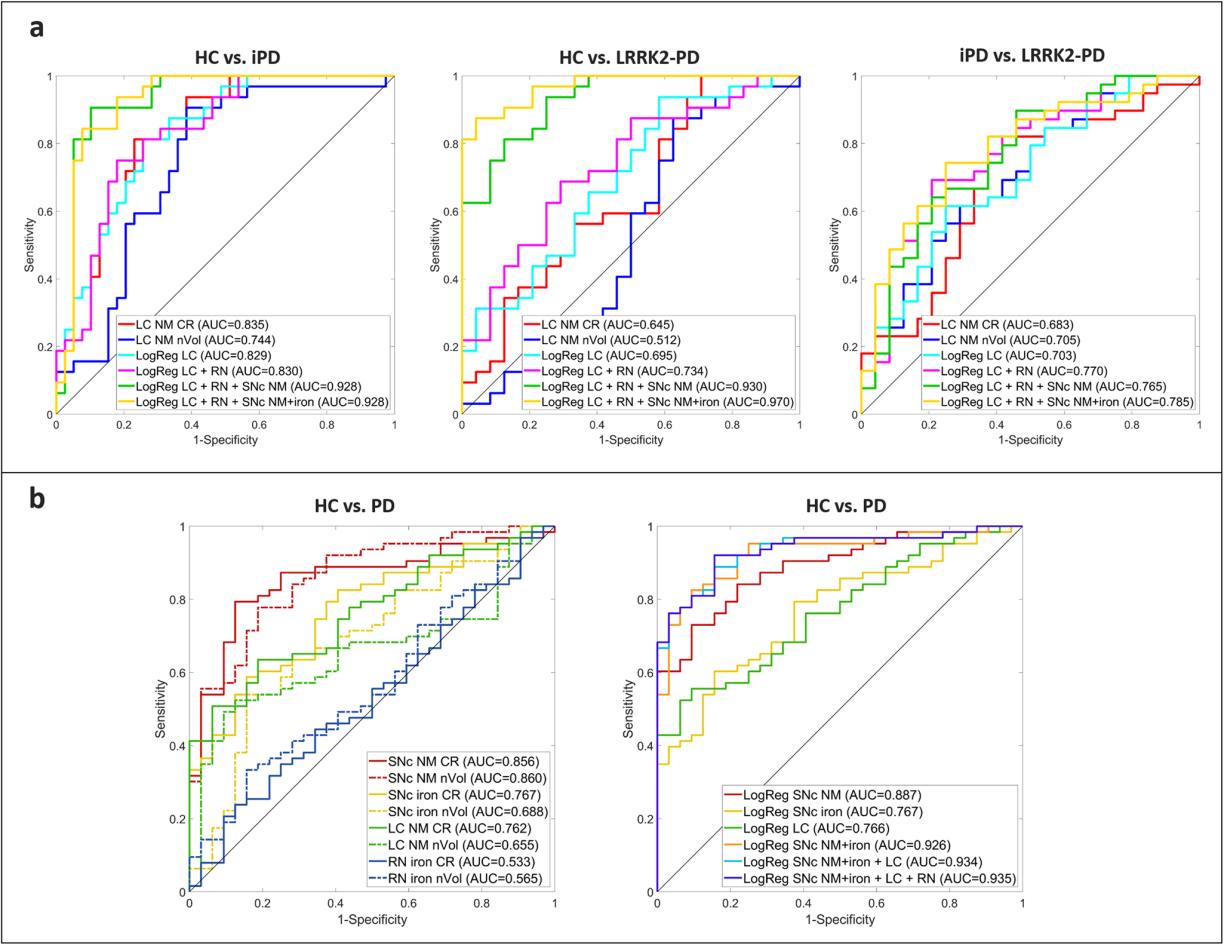
ROC analyses for 3D-ASBP quantitative image variables’ diagnostic performance. **a** Pairwise comparison for the differentiation of HC, iPD, and LRRK2-PD groups. **b** HC vs. PD group differentiation using individual quantitative parameters (left) or combined parameters through logistic regression models.

All groups (i.e., HC, iPD, and LRRK2-PD) were first compared pairwise using the AUC values of their respective ROC curves (Fig. 6a, Supplementary Table 4). The analysis showed a moderate-to-high discrimination power of the LC, ranging from AUCs of 0.7 (HC vs. LRRK2-PD, or iPD vs. LRRK2-PD), to 0.83 (HC vs. iPD). The addition of RN to the model provided a 10% increase in the discrimination power between iPD and LRRK2-PD (from 0.70 to 0.77 AUC), whereas the addition of SNc NM and SNc iron to the model, further increased the discrimination power of the method for all groups. The complete model achieved AUC values of 0.93 for HC vs. iPD, 0.97 for HC vs. LRRK2-PD, and 0.79 for iPD vs. LRRK2-PD.

The eight MRI quantitative variables (SNc NM CR and nVol, SNc iron CR and nVol, LC NM CR and nVol, and RN iron CR and nVol) were then used to assess the diagnostic performance for HC vs. all PD subjects (Fig. 6b, Supplementary Table 4). The variables were first evaluated individually (Fig. 6b, left) and secondly, combined through binary logistic regression to incorporate different models of increasing complexity (Fig. 6b, right). SNc NM is the best discriminator between HC and PD (AUC = 0.89), followed by LC NM (AUC = 0.77) and SNc iron (0.77). RN iron did not discriminate between these groups. When NM and iron in the SNc were combined, the AUC raised up to 0.93, and when the LC NM and RN iron parameters were added to the model, the highest AUC (i.e., 0.94) was achieved for the HC vs. PD comparison.

### Quantitative and diagnostic analysis of early-stage PD

In order to investigate the potential of the 3D-ABSP for NM and iron measurement in PD early disease stages, a sub-cohort of PD patients with <5 years of PD symptoms was analyzed (*n* = 18; 11 iPD, 7 LRRK2-PD). The analysis of all early-stage PD patients compared with healthy controls showed that all NM parameters (SNc NM CR and nVol, and LC NM CR and nVol) were statistically significantly lower in early-stage PD than in HC (corrected *p* < 0.001). In addition, SNc iron CR was higher in early-stage PD, whereas no statistically significant differences were found neither for SNc iron nVol nor for RN iron CR and nVol (Supplementary Fig. 6a–h). Subsequent ROC analyses confirmed that SNc NM and LC NM were the best discriminators between HC and early-stage PD (AUC = 0.87 for SNc NM, and AUC = 0.80 for LC NM). In contrast, SNc iron achieved an AUC of 0.70, and RN iron did not discriminate between these groups (Supplementary Fig. 6i–j). Combining SNc NM and iron the AUC raised up to 0.88 and, when all the quantitative brainstem MRI measures were included in the model, the AUC boosted to 0.92 (Supplementary Fig. 6j).

### Association between brainstem MRI parameters and demographic and clinical variables in PD

Multiple regression analyses indicated that four out of the eight MRI brainstem parameters were consistently predicted by demographic and clinical variables in PD patients (Supplementary Results, Multiple regression analyses of MRI brainstem measures, Supplementary Fig. 7, and Supplementary Table 5). Specifically, a decrease in SNc NM CR was related to the presence of psychiatric symptoms. An increase in SNc iron CR and SNc iron nVol was also related to longer disease duration, whereas a decrease in SNc iron nVol was associated with older age. Finally, a decrease in LC NM nVol was associated with the male sex.

## Discussion

In this study, we quantified and anatomically mapped the loss of NM and iron burden in the midbrain structures of PD patients and healthy controls using a highly efficient 3D-ABSP applied to MRI sequence analysis. Our automatic unbiased measurements of NM and iron revealed precise signatures for LRRK2-PD in SNc, LC, and RN (Table 2). A pronounced NM loss was found in the ventrolateral tier of the SNc of both idiopathic and monogenic PD patients. While the SNc was a good discriminator between PD patients and controls, the LC was the structure that best discriminated among PD subgroups: LRRK2-PD had the LC NM content preserved, whereas iPD showed a substantial LC NM reduction.

Firstly, our results showed, a loss of NM in terms of NM content and volume (both CR and nVol) in the SNc in all PD patients, that followed a spatial neurodegeneration pattern (lateral ventral to medial ventral and dorsal SNc tiers) that showed the main impairment in areas responsible for the putaminal output, which are involved in the impaired motor function in PD. Previous histopathological studies have found an aging-related loss of SNc-pigmented neurons starting in the dorsal tier followed by the medial ventral tier, whereas PD brains showed a different neurodegenerative pattern: NM loss starts in the lateral ventral tier and is followed by medial ventral and dorsal tiers^4^. In addition, we found that in LRRK2-PD the NM SNc loss was similar to iPD, confirming the results of a previous study in which SNc neuronal dopaminergic loss among LRRK2 mutation carriers was similar to sporadic PD as described also for the motor features^17^.

One striking result of the current study was the preservation of LC NM in LRRK2-PD, as opposed to the NM loss of this structure in iPD. Very importantly, disease duration did not correlate either with the LC NM CR or the nVol in LRRK2-PD, thus reinforcing a real NM preservation in LC in LRRK2-PD despite the disease progression. These results are consistent with the different mRNA expression profiles found in LC for LRRK2-PD and iPD^18^ and can explain the low prevalence of cognitive impairment and RBD found in G2019S *LRRK2* patients^19^. These findings are in accordance with the relative LC preservation in brains from *I2020T* LRRK2-PD carriers^20^. Thus, these results strongly suggest that LC preservation is a valid imaging signature for G2019S *LRRK2* PD.

We also found an asymmetrical left brain predominance of NM nVol in SNc and LC in both controls and patients that could be related to handedness, confirming the results of a previous study^21^. Further studies are needed to further ascertain whether the SNc and LC left/right asymmetry have an effect on the PD phenotype by interacting with other factors such as handedness or disease onset laterality.

It has been proposed that the ferric iron load accelerates α-synuclein aggregation^22^, and an increase of iron levels plus a decrease of SN ferritin can contribute to nigral degeneration^23^. SN and RN iron concentrations measured with quantitative susceptibility mapping show a nonlinear quadratic relationship with aging in controls^24^. An increased iron burden has been found in early PD stages in the SN and advanced disease stages in the RN^25^. In agreement with this evidence, we have found that SNc iron CR is similar to that of the SNr in PD patients, whereas, in HC, SNc iron CR was lower than in the SNr, confirming the already described physiological pattern of iron burden^26^. The iron measures within SNc showed a higher iron burden in PD patients than controls, specifically among iPD (CR) and LRRK2-PD (CR and nVol). Although the role of human RN and its relation to the dysfunctional basal ganglia in PD is not known, RN has been functionally connected to the basal ganglia cerebellum and cerebral cortex for the execution of complex tasks, tremors, and stereotyped movements^27^. The functional effects of the increased iron burden in RN among LRRK2-PD are difficult to interpret, but it could be related to an increased LRRK2 expression in the nuclear envelope of RN and other brain structures^28^.

A statistically significant interaction in the factorial analyses confirmed an opposite behavior of NM and iron measurements in the SNc: all PD groups showed a NM CR and nVol reduction and an increase of iron CR and nVol when compared with HC. In all PD groups, the topographical distribution of NM and iron in SNc anatomical quadrants revealed a more pronounced NM loss in the ventrolateral *tier* (i.e., quadrants Q_1_ and Q_2_; Fig. 3), and the disappearance of the NG-1, the main putamen output, consistently with previous pathological studies^29,30^. Considering that the PD patients included in our study were in moderate HY stages, and most PD subgroups had similar disease duration, they showed different SNc NM nVol loss (25%, and 21% for iPD and LRRK2-PD groups, respectively). In all PD patients, the iron burden was increased in the whole SNc, except for Q_1_ (ventral SNc), which was the region with the highest iron burden in both patients and controls. The probabilistic maps of NM and iron content in the SNc for HC and PD visually confirmed all these findings along with the disappearance of NG-1 (corresponding to Q_2_ and Q_4_) and NG-2 (corresponding to Q_3_) in PD, in accordance with previous studies^31–33^. In addition, SNc iron and NM nVols were asymmetrical with left-side predominance in HC and all PD subgroups (Supplementary Fig. 2).

ROC analyses of SNc variables indicated that NM measurements were better discriminators between controls and PD patients than the iron measurements. These results suggest that SNc NM loss could be a better indicator than iron content for monitoring PD progression, in agreement with pathological studies^3^. The combined discriminative power of SNc NM, SNc iron, and LC NM achieved an AUC>0.92 for every pairwise comparison among HC, iPD, LRRK2-PD, except for the comparison between iPD and LRRK2-PD (AUC = 0.79). These results further support that these variables reveal specific MRI signatures for LRRK2-PD that differentiate them from iPD and HC. In addition, an AUC of 0.94 was achieved between HC and all PD patients, in spite of the heterogeneity of the PD sample and high percentage of monogenic PD included in our study. The review of the literature shows that only DATscan achieves a discriminative AUC higher than the one obtained by our 3D-ABSP for the PD diagnosis. NM-MRI is a promising technique, with potential advantages over DAT imaging: no need for radiotracer injection, lower costs, and faster acquisitions. In addition, to date, no neuroimaging biomarkers have been found for monogenic PD^34^.

The analysis of a sub-cohort of 18 patients in early PD staging (<5 years of disease) suggested that NM is a better marker than iron for early PD. Both SNc NM and LC NM maintain the discrimination power between HC and early-stage PD patients at the same level as the one found between HC and the entire PD cohort, whereas only SNc iron CR showed statistically significant differences between HC and early-stage PD groups. A logistic regression model combining SNc iron parameters showed a decrease in the diagnostic performance when considering only the early-stage PD group -from an AUC of 0.77 for the entire PD cohort, to an AUC of 0.70-. These results suggest that iron slowly accumulates in the SNc as the disease progresses, whereas a more pronounced NM loss is already found at the initial stages of the disease. Nevertheless, a combined logistic regression model including all NM and iron parameters achieved an AUC of 0.92 between HC and early-stage PD, almost identical to the one obtained after comparing HC with the entire PD cohort (i.e., 0.94), which suggests that our 3D-ABSP has a strong potential as an early PD diagnosis tool. Further studies including a larger early PD sample will have to be carried out to further investigate the potential of NM and iron MRI measurements in discriminating idiopathic and monogenic PD at the early stages of the disease.

Brainstem measurements of the SNc in the entire cohort of PD patients were associated with disease duration. Specifically, SNc iron CR and nVol were increased proportionally to disease duration. The association between SNc iron CR/nVol and disease progression found is consistent with previous investigations^35^. These results pointed out that the SNc NM loss cooccurs with the increase of iron load over time and supports the hypothesis by which iron metabolism dysfunction in the SN may accelerate neurodegeneration in PD^10,31^.

We found a decrease in LC NM nVol associated with being male among the PD population. We suggest that further studies are needed in order to test whether this potential neuroprotective effect observed in females is linked to hormonal effects since it has been suggested that estrogens may increase the survival of nigrostriatal noradrenergic neurons^36^.

Our findings highlight the relevance of NM and iron MRI analysis as imaging markers for PD diagnosis. However, we recognize that some limitations can affect some of the results. Firstly, the lack of left-handed subjects made it difficult to interpret the asymmetry found in the SN and LC. Secondly, the SWI sequence used may be prone to image artifacts due to the orientation-dependence of the phase information, and most studies that quantify iron content currently use quantitative susceptibility mapping (QSM) instead^37^. Although with SWI we do not obtain absolute quantitative iron concentration values, we normalized the intensities and calculated CR and nVol measures that allowed a fair comparison of iron content between HCs and PD subtypes. And thirdly, since we screened only the two main PD-related genes, we are aware that the inclusion of a group of *GBA, PARKIN*, or *PINK1* PD mutation carriers could also increase the knowledge of neuroimaging behavior in other monogenic PD patients.

We have proved the potential of 3D-ABSP to become a diagnostic tool for the characterization and follow-up of PD based on imaging of brainstem structures. In terms of clinical applicability, we conclude that the specific imaging patterns found could be used as neuroimaging biomarkers for PD diagnosis and severity. Furthermore, they can also be used to differentiate specific PD genetic subgroups. Nevertheless, we are aware that it would be convenient for future studies to reproduce our findings to validate the role of NM and iron neuroimaging as PD biomarkers.

## Methods

### Sample size estimation

We calculated the optimal sample size of the corresponding expected effect size^38^ with G*Power 3.1.9.6 software. Based on our previous study^15^, we expected large effect sizes in the normalized volume of the SNc in the NM-MRI sequence comparing patients with controls (*d* = 1.20). Thus, the optimal sample size obtained for the expected effect size was 32 participants (with standard values *α* = 0.05, power = 0.8, one-tail tests).

### Subjects

PD Patients fulfilled the UK Brain Bank Parkinson’s disease criteria^39^. In addition, patient selection was carried out considering their genetic status in order to enrich the genetically-determined PD groups and controls to match sex and age with them. HC individuals with no neurological disorders were recruited among their spouses. Written informed consent was obtained from all subjects, and the study was approved by the University of Navarra Research Ethics Committee.

### Neurological and neuropsychological assessment

Patients underwent an interview covering demographic data, family history of neurological diseases, the *Mini-Mental State Examination* (MMSE)^40^, the *Parkinson Disease-Cognitive Rating Scale battery* (PD-CRS)^41^, the *Unified Parkinson’s Disease Rating Scale* (UPDRS-III)^42^, *Modified Hoehn and Yahr Scale* (HY)^43^, the Non-Motor Symptoms Scale (NMSS)^44^, and *Edinburgh Handedness Inventory*^16^. Levodopa equivalent daily dose (LEDD)^45^ was also calculated. Disease onset was established as the age when the Parkinsonian motor signs started self-reported when possible or reported by the caregiver. In addition, other clinical variables were assessed: “psychiatric symptoms” when depression or other psychiatric conditions were present, “hyposmia” (from NMSS item 28), and “other sleep disorders” (from NMSS items 3 and 5). RBD was recorded with the *REM Sleep Behavior Disorder Single-Question Screen*^46^. The UPDRS-III was assessed in an ON state right before the MRI scan.

### Genetic analysis

Genomic DNA was isolated from leukocytes. Patients were screened for *LRRK2* p.G2019S mutation by a custom-designed allele-specific PCR Taqman® assay and *GBA* gene analysis long-range PCR with confirmatory Sanger sequencing was performed^47^.

### Neuroimaging analysis

#### MRI protocol and acquisition

Levodopa dosing was rescheduled in 22 patients to avoid dyskinesia during MRI scan. MRI scans were carried out on a 3-T MAGNETOM Skyra MRI scanner (Siemens Healthineers, Erlangen, Germany), using a 32-channel head array during a 32-min session for anatomical acquisition, 3D NM-sensitive sequence, and susceptibility-weighted images (SWI) datasets. The anatomical T1-weighted image was acquired with an MPRAGE sequence of 5 min. The following imaging parameters were employed: 1 mm-isotropic resolution, field of view (FOV) = 256 × 192 mm^2^, matrix = 256 × 192 voxels, 160 axial slices, repetition time (TR)/echo time (TE) = 1620/3.09 ms, Inversion Time = 650 ms, flip angle = 15º. The NM-sensitive sequences (i.e., NM-MRI) were obtained with a 3D-NM-sensitive T1-weighted turbo spin-echo sequence^48^ with the following parameters: repetition time/echo time, 34/4.91 ms; flip angle = 20º; 40 slices, 1 mm slice thickness, 0.2-mm gap, 512 × 408 acquisition matrix, 220 × 175 FOV, (voxel 0.6 × 0.6 × 1.0 mm), bandwidth 190 Hz/pixel, four averages. The SWI sequences (i.e., iron-MRI) were obtained by combining a long-TE high-resolution fully flow-compensated three-dimensional (3D) GRE sequence with filtered phase information in each voxel^49^ with the following parameters: TR/TE, 24/34 ms, flip angle = 10°, 22 cm FOV, 256 × 512 acquisition matrix, and 2 mm slice thickness (voxel-size 0.7 × 0.7 × 2.0 mm).

#### Image preprocessing

The slices were oriented orthogonally to the fourth ventricle floor and covered from the posterior commissure to the pons. Four excitations in the NM sequence and three excitations in the iron sequence were acquired and realigned offline to correct head movement. This image preprocessing was performed using SPM12 (Wellcome Trust Center for Neuroimaging, London, UK; http://www.fil.ion.ucl.ac.uk) and custom scripts in Matlab R2021a (Mathworks, MA, USA). In order to facilitate and standardize manual delineations, MR images were manually reoriented to adapt them to the orientation of the axial and mid-sagittal plane of a canonical T1 template image in SPM8.

#### Automatic image segmentation of brainstem structures

All the brainstem structures of interest were automatically segmented from the NM-MRI and iron-MRI sequences using the 3D-ABSP (Fig. 1). For this purpose, we created two static atlases of brainstem structures, one for the NM-MRI sequence and another one for the iron-MRI sequence, consisting of images of 32 HC and their corresponding manual annotations. The manual annotations were produced by an experienced neurologist (MAP), who delineated the structures of interest (SNc, LC, and brainstem in the NM-MRI sequences; whole iron deposit in SN, RN, and brainstem in the iron-MRI sequences) from all slices of the images of the 32 HC (Supplementary Fig. 8). Manual segmentation was blinded to the different groups, including controls.

The automatic segmentation of NM and iron-rich brainstem structures involved a multiresolution, three-step image registration of the target image with each of the atlas images, followed by a label fusion strategy to generate the final segmentation masks^15^. As quality-control of the segmentation, all brainstem structures of the 32 HC were segmented following a leave-one-out strategy (i.e., using an atlas elaborated from the remaining 31 HC), and the average Dice Similarity Coefficient (DSC) between the segmentation masks and their corresponding manual annotations for each brainstem structure was calculated as a quality-control segmentation accuracy score. After the quality of the segmentation was confirmed, the 71 PD images were segmented using the entire atlas composed of the images of the 32 HC.

#### Intra-subject spatial alignment of NM and iron sequences

Each subject’s individual NM and iron MRI images were aligned using a multiresolution rigid registration algorithm implemented with Elastix^50^. To assess the quality of the alignment, the DSC between the brainstem manual annotations of the original NM image and the transformed iron image was calculated.

#### Quantification of NM and iron in brainstem structures

The labels produced by the automatic segmentation of brainstem structures were used to quantify the amount of NM in SNc and LC, and the amount of iron in SNc and RN. Note that the intra-subject accurate alignment of both sequences allows applying the SNc segmentation mask obtained from the NM image to the iron image and constrains the iron measurement to the SNc. We measured the contrast ratio (CR), and the volume of each brainstem structure which was normalized to the total volume of gray matter (nVol). The gray matter volume was estimated with the *get_totals* function in Matlab. The CR was defined as the relative increase of the average intensity of the structure compared with the average intensity of the brainstem (normalized brightness). The automatically segmented structures were thresholded to measure the CR and nVol from hyperintense voxels in the case of NM^15^, and from hypointense voxels in the case of iron (Supplementary Figs. 9 and 10). CR values of iron were inverted in sign to facilitate interpretation (i.e., higher CR is translated into darker regions in the case of iron, whereas it means the region is brighter in the case of NM).

NM-iron interactions in the SNc were analyzed in-depth following an inter-subject image alignment strategy to create NM and iron spatial probabilistic maps and to display their specific spatial distribution patterns in the SNc. The topographical distribution of NM and iron content in this structure was thoroughly analyzed by fitting an elliptical-section cylinder which divided the SNc into four anatomical quadrants: Q_1_ corresponding to the ventral, Q_2_ mainly to the lateral, Q_3_ to the medial-rostral, and Q_4_ to the medial-caudal SNc (Fig. 1, Supplementary Fig. 11). All calculations were performed in the subject space.

#### Probabilistic maps of NM and iron content in brainstem nuclei

A reference HC image of the NM sequence with the highest DSC after the SNc segmentation was selected as the reference. Then the remaining NM sequence images were registered to the reference image following the same three-step registration framework described above.

Similarly, all the iron sequence images that were previously registered to their corresponding NM sequence image, underwent the same transformation to align them to the reference NM image. All brainstem structures’ segmentation masks underwent the same transformation as well. Hence, all the NM and iron sequence images were transformed into the same coordinate space, which allowed the creation of NM and iron content probabilistic maps using the segmentation masks of brainstem structures. Each voxel in the probabilistic map contained the sum of the segmentation masks of all the images (i.e., ‘1’s where the segmented structure of interest is present and ‘0’s where it is not) normalized to the number of subjects in the group for which the map was created (in the SNc *N* = 32 for HC and *N* = 63 for PD; in the LC *N* = 32 for HC, *N* = 39 for iPD and *N* = 24 for LRRK2-PD).

#### Spatial distribution of NM and iron in the SNc

A refined spatial analysis of the NM and iron content in the SNc was carried out by measuring the contrast ratio (CR) and normalized volume (nVol) in each anatomical quadrant and axial slice of the structure. To this end, NM and iron sequence images were aligned to the reference NM image as depicted in the previous section. Then, an elliptical-section cylinder was optimally fitted to the SNc, dividing the structure into four distinct anatomical quadrants. Q_1_ corresponded to the ventral SNc, Q_2_ mainly to the lateral SNc, Q_3_ to the medial-rostral SNc, and Q_4_ to the medial-caudal SNc. The automatic 3D segmentation was used to mask each quadrant in the elliptic cylinder, and CR and nVol per quadrant and slice were calculated for NM and iron signals (Supplementary Fig. 11).

### Statistical analyses

Considering the distribution of the population studied, non-parametric (robust) statistical analyses were employed for group comparisons. One-way robust ANOVAs (WRS2 R package^51^; trimming value = 0.2) were run to examine group differences in the CR and nVol of brainstem structures in each sequence. Two-way robust mixed ANOVAs were used to evaluate the combined effects of group and region, group and side, and group and sequence. Post hoc *t* tests from the bootstrap version (number of samples = 500) were used. The effect size of post hoc comparisons was evaluated with a robust, heteroscedastic generalization of Cohen’s *d*^52^. The confidence intervals and the *p* values were adjusted for multiple testing^53^. Potential associations between brainstem MRI measures and demographic and clinical variables for the entire cohort of PD patients were evaluated using robust multiple regression analyses with MASS R package^54^. Robust multiple regression analyses with Huber M estimator (MASS R package) were applied to predict each of the brainstem MRI measures from demographic and clinical variables in PD subjects (i.e., age, sex, handedness, most affected side, years of education, disease duration, H&Y, UPDRS-III, LEDD, MMSE, PD-CRS, and presence of psychiatric disorders, hyposmia, RBD, or other sleep disturbances). To consider the amount and the interdependent nature of predictors, meaningful variables for each MRI parameter were obtained by means of feature selection with the Boruta algorithm^55^. Finally, robust regression analyses were repeated to predict each MRI brainstem measure from the meaningful variables suggested by the feature selection algorithm. Multiple-constraint hypotheses were conducted using robust Wald tests. Finally, the diagnostic performance of the 3D-ABSP was assessed by means of binary logistic regression analyses in Matlab. Bootstrapping with 1000 iterations was used to obtain AUC confidence intervals with 95% level of confidence. All tests were two-tailed, and *p* values <0.05 (corrected for multiple comparisons) were considered statistically significant.

### Reporting summary

Further information on research design is available in the Nature Research Reporting Summary linked to this article.

## Supplementary information

Supplemental material

Consortium description

Reporting Summary

Code compressed file

## Data Availability

Written requests for access to the data reported in this paper will be considered upon request to the corresponding author M.A.P., C.O.S., and P.P. as long as the appropriateness of the use of data exists. If the use is appropriate, a data-sharing agreement will be signed before a fully de-identified version of the dataset used for analysis is available.

## Code availability

The Matlab scripts used for data analysis are available in the supplementary information.
