# Supplementary material for: Brainstem neuromelanin and iron MRI reveals a precise signature for idiopathic and LRRK2 Parkinson’s disease
Source: NPJ Parkinsons Dis. 2023 Apr 15;9:62. doi: 10.1038/s41531-023-00503-2 (PMC10105708; doi:10.1038/s41531-023-00503-2)
Supplement: Supplementary file 1 — Supplemental material [file 41531_2023_503_MOESM1_ESM.pdf]

Brainstem neuromelanin and iron MRI reveals a precise signature for idiopathic and LRRK2 Parkinson's Disease

SUPPLEMENTARY RESULTS

Automatic segmentation and image alignment quality-control scores

NM-MRI images were used to segment SNc and LC using the 3D-ABSP automatically. Quality-control segmentation accuracy scores (DSCs) were 0.89, 0.79, and 0.60 for the brainstem, SNc, and LC, respectively, confirming a high degree of segmentation accuracy, given the complexity of the task, the resolution of the images, and the size of the segmented structures. The CR and nVol of the SNc and LC were next quantified for both HC and PD groups.

SWI images of all subjects were used to automatically segment the iron deposits in the SN and the RN. The quality-control DSC values were 0.87, 0.79, and 0.81 for the whole brainstem, SN and RN, respectively, reflecting again a high degree of segmentation accuracy. The automatic segmentations were then used to quantify the iron CR and nVol of these structures.

NM and SWI images were aligned intra-subject to allow the study of NM and iron interactions in the brainstem structures. A quality control DSC value of 0.93, obtained between manual annotations of the brainstem of the original NM images and transformed iron images, reflected optimal intra-subject alignment accuracy.

Multiple regression analyses of MRI brainstem measures

Results of the robust regression for the SNc NM CR indicated that the presence of psychiatric symptoms explained 9.1% of its variability. Specifically, a decrease in SNc NM CR was determined to be related to psychiatric symptoms (Supplementary Fig. 7a). For SNc iron CR, regression analyses determined a positive association with disease duration (Supplementary

Fig. 7b). Regression analyses for the SNc iron nVol indicated that age and disease duration significantly predicted 28.4% of the variability. Specifically, it was found that an increase in SNc iron nVol was associated with lower age (Supplementary Fig. 7c), and with a longer duration of the disease (Supplementary Fig. 7d). The statistically significant negative association between SNc iron nVol and age obtained in PD patients (Spearman's rho=-0.32, p=0.002, bootstrap p-values adjusted for multiplicity using Holm's method) was not observed in HC (rho=-0.22, p=0.16). When considering the PD subgroups, this association was only significant in the case of iPD (rho=-0.33, p=0.001), with no significant associations in LRRK2-PD (rho=-0.23, p=0.41). Thus, although the association between SNc iron nVol and age is notable for the entire cohort of patients, this effect is determined by the influence of iPD patients.

For the LC NM nVol, sex was found to predict 3.4% of its variability. Lower LC NM nVol was associated with being male (Supplementary Fig. 7e). The significant association between LC NM nVol and being male obtained in PD patients (logistic regression analysis, p=0.012, bootstrap p-values adjusted for multiplicity using Holm's method) was not observed in HC (p=0.59). When taking into account the subgroups, there was statistical significance in iPD (p<0.001), but there was no significant association in LRRK2-PD (p=0.73). In summary, although the association between LC NM nVol and sex is significant in the sample cohort, this association was due to the influence of iPD.

SNc NM nVol, LC NM CR, RN iron CR, and RN iron nVol were not found to be significantly predicted by any demographic or clinical variable. The model statistics of regression analyses for MRI brainstem measures with all predictors, the meaningful variables for each model, and the regression analyses statistics for the significant predictors are detailed in Supplementary Table 5.

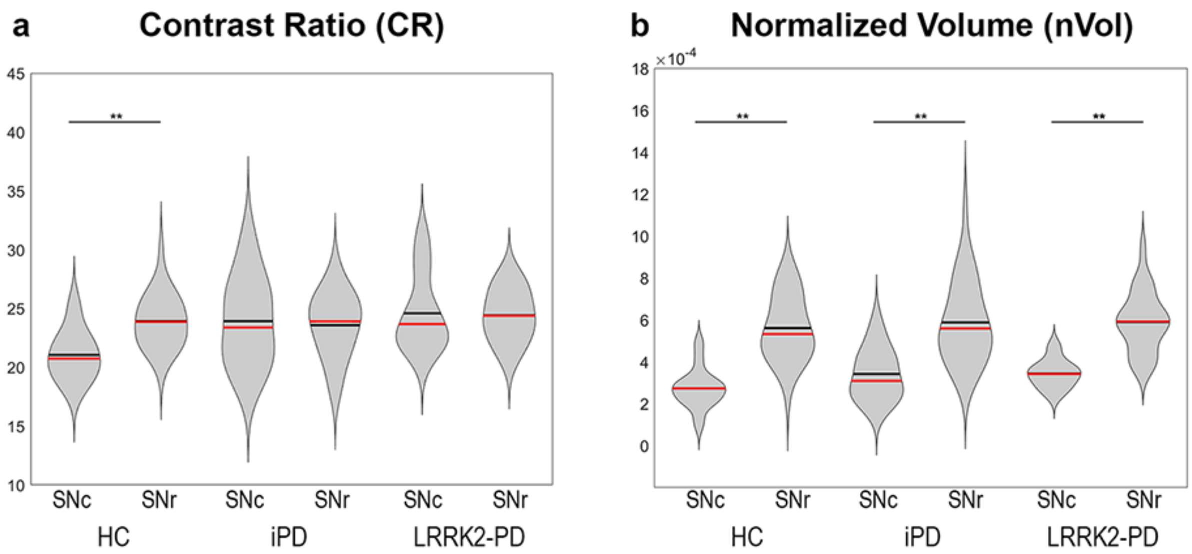

Supplementary Fig. 1. Quantification of iron in SNc vs. iron in SNr in all groups. a Contrast Ratio, and **b** Normalised Volume. A two-way mixed ANOVA was performed in order to assess group and region effects as well as group x region interactions in iron CR and nVol. For the CR, we found a main effect of region ($F=18.7$, $p<0.001$, SNr > SNc) and a group x region interaction ($F=12.41$, $p<0.001$). For the nVol, a main effect of Region was found ($F=277.38$, $p<0.001$, SNr > SNc) (* $p < 0.05$, ** $p < 0.001$, corrected for multiple testing).

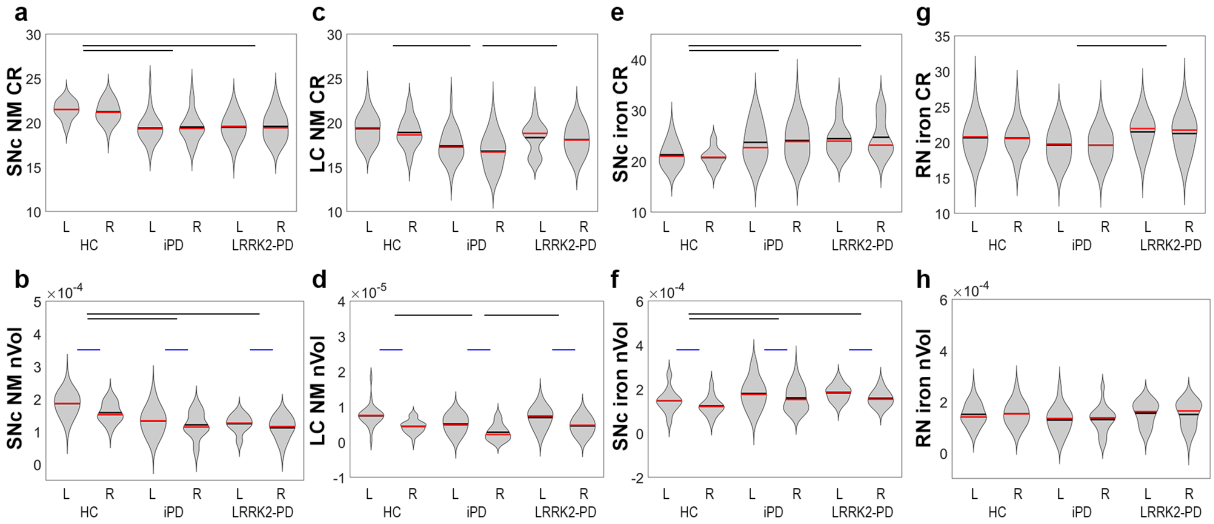

Supplementary Fig. 2. Violin plots of CR (top row) and nVol (bottom row) of the brainstem structures: SNc (**a-b**) and LC (**c-d**) in the NM sequence, and SNc (**e-f**) and RN (**g-h**) in the iron sequence. In the violin plots, red lines represent the median, and black lines represent the mean. Statistically significant brain lateral asymmetry (left vs. right) of the structures is shown with blue horizontal lines, and significant group differences are shown with grey horizontal lines.

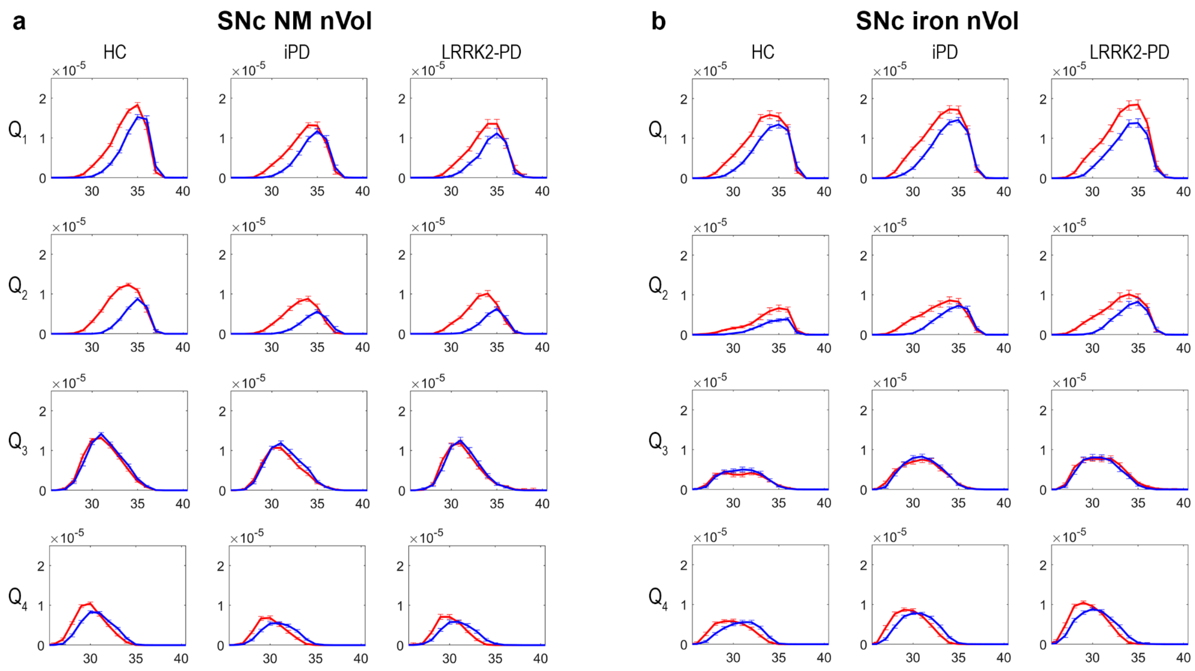

Supplementary Fig. 3. Distribution of NM nVol (**a**) and iron nVol (**b**) in left and right SNc by group, anatomical quadrant, and slice (from caudal to dorsal). Red=Left SNc, Blue=Right SNc.

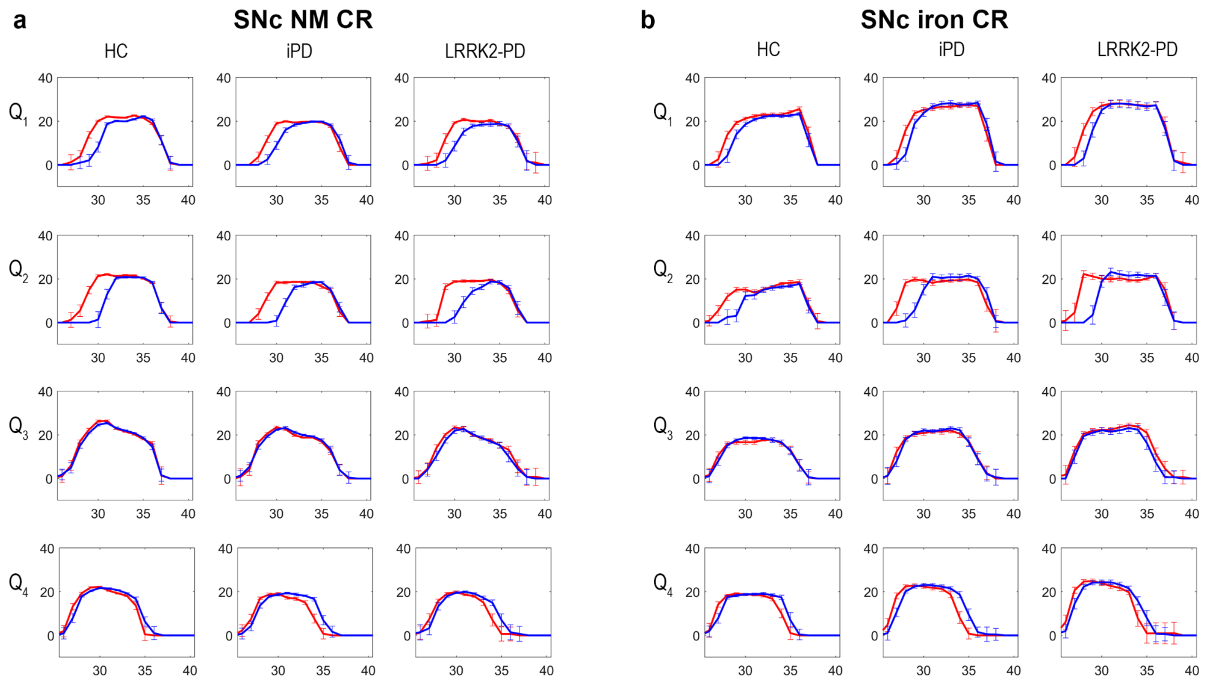

Supplementary Fig. 4. Distribution of NM CR (**a**) and iron CR (**b**) in left and right SNc by group, anatomical quadrant, and slice (from caudal to dorsal). Red=Left SNc, Blue=Right SNc.

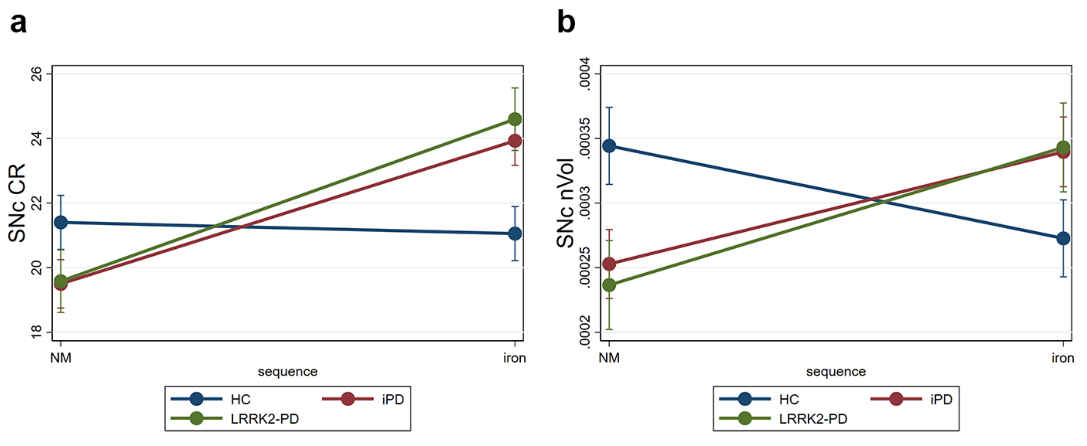

Supplementary Fig. 5. Group × sequence interactions in **a** CR and **b** nVol of the SNc between HC and PD subgroups.

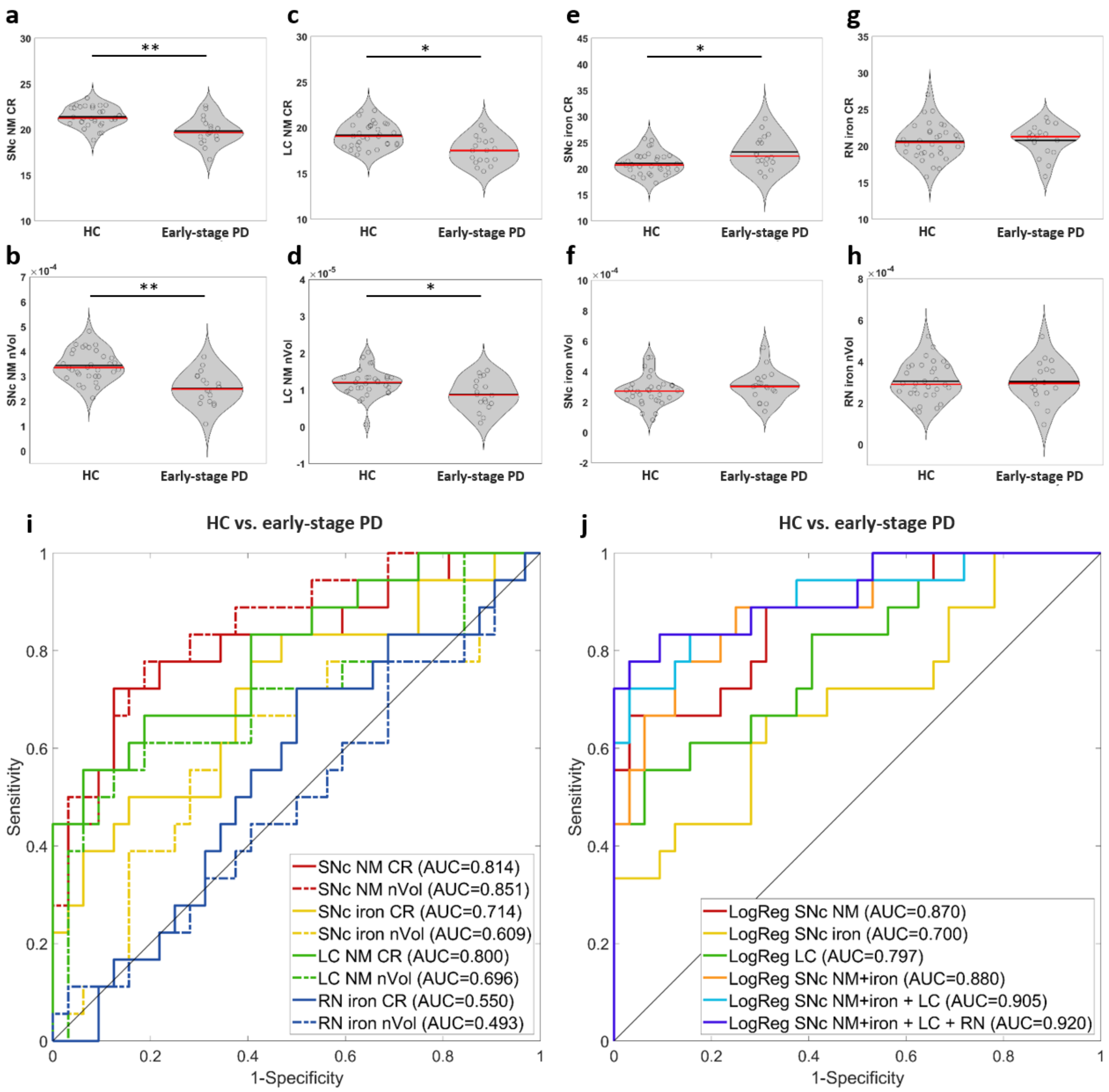

Supplementary Fig. 6. Quantitative and diagnostic analysis of early-stage PD in a sub-cohort of 20 PD patients with less than 5 years since their diagnosis. Violin plots of the quantitative brainstem MRI measures: **a** SNc NM CR, **b** SNc NM nVol, **c** LC NM CR, **d** LC NM nVol, **e** SNc iron CR, **f** SNc iron nVol, **g** RN iron CR, and **h** RN iron nVol. ROC curves of HC vs. early-stage PD differentiation using: **i** Individual quantitative brainstem MRI measures; and **j** Combined parameters through binary logistic regression models.

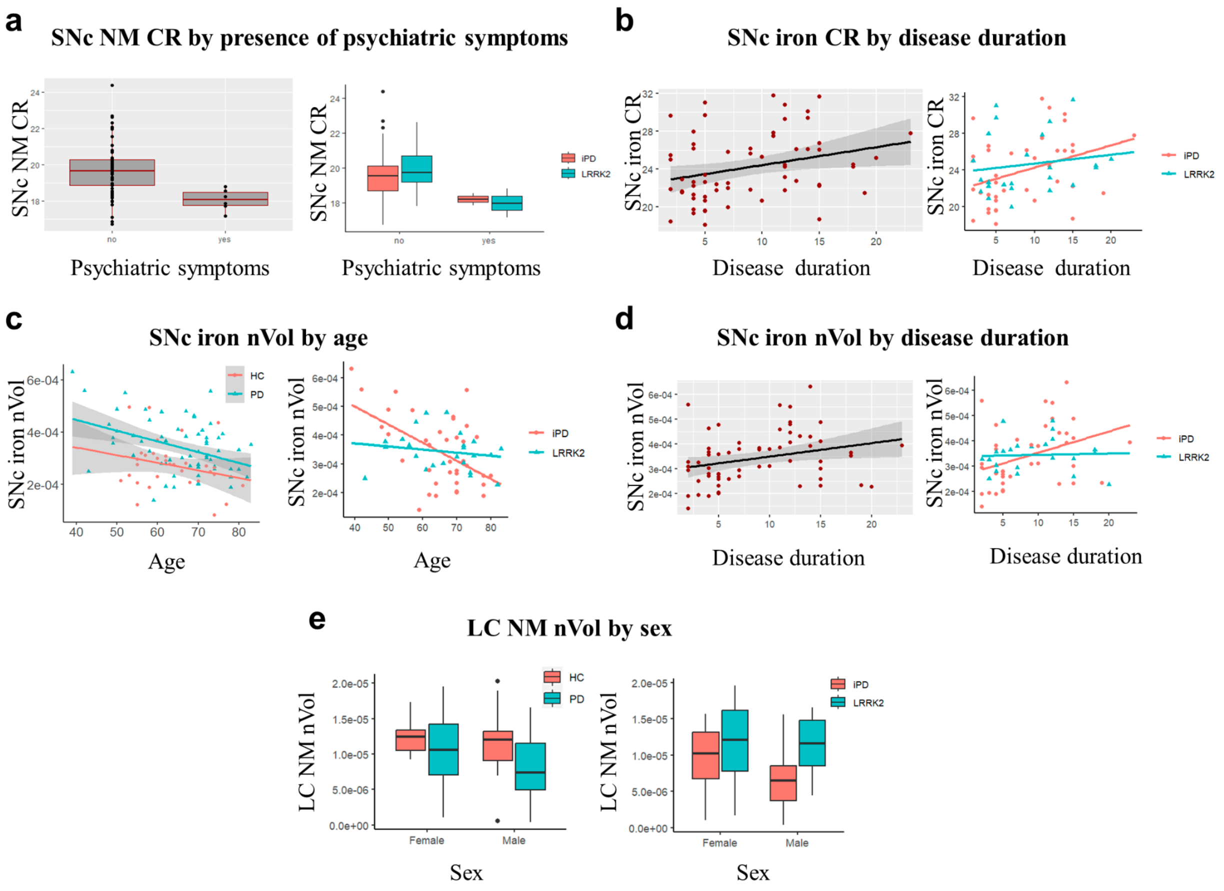

Supplementary Fig. 7. Associations between brainstem MRI measures and demographic and clinical variables in PD patients. Although the statistically significant associations were calculated in the entire PD cohort (Supplementary Table 5), linear models and boxplots are depicted by PD subtypes to facilitate visual inspection of subgroups' behaviour. **a** SNc NM CR and the presence of psychiatric symptoms; **b** SNc NM CR and the disease duration; **c** SNc iron nVol and age; **d** SNc iron nVol and disease duration; **e** LC NM nVol and sex.

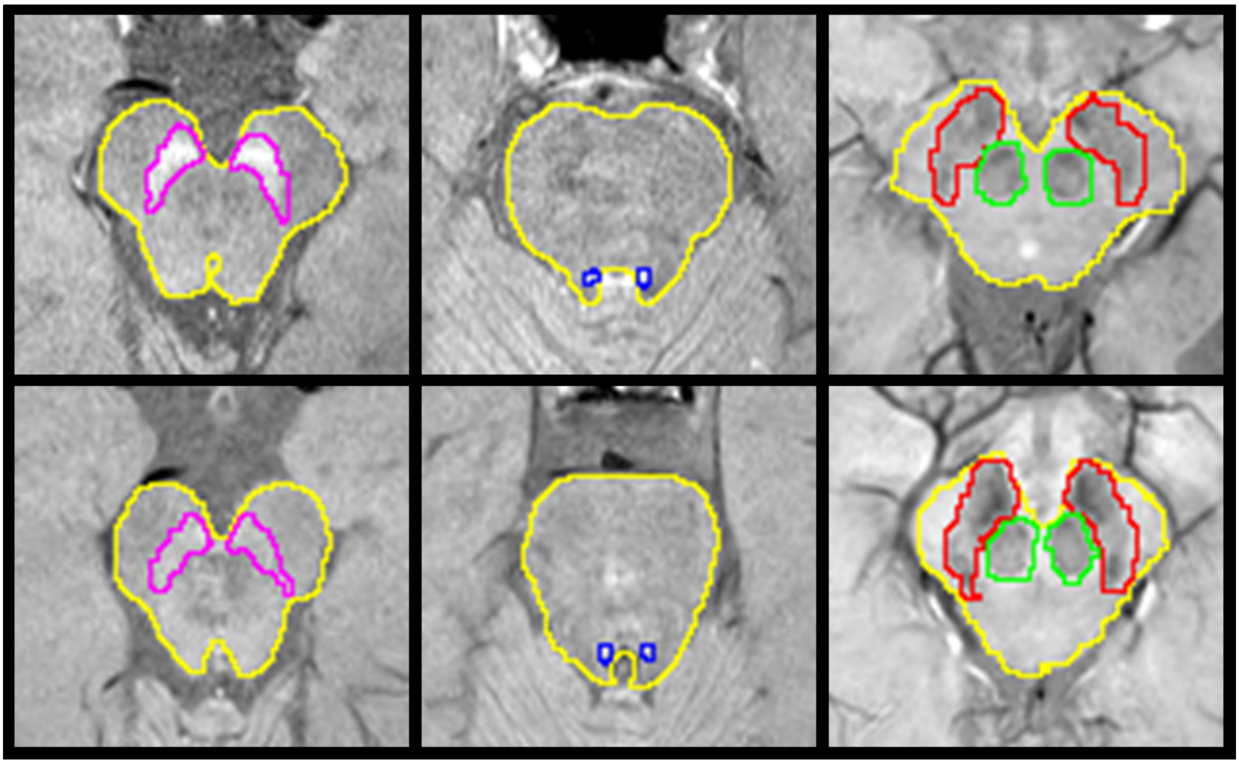

Supplementary Fig. 8. Example images of manual segmentation of brainstem structures in one HC (top row) and one PD patient (bottom row). Brainstem limits are annotated in yellow, SNc in magenta (left column) and LC in blue (middle column) in the NM sequence, and the iron deposit in SN in red and the RN in green (right column) in the iron sequence.

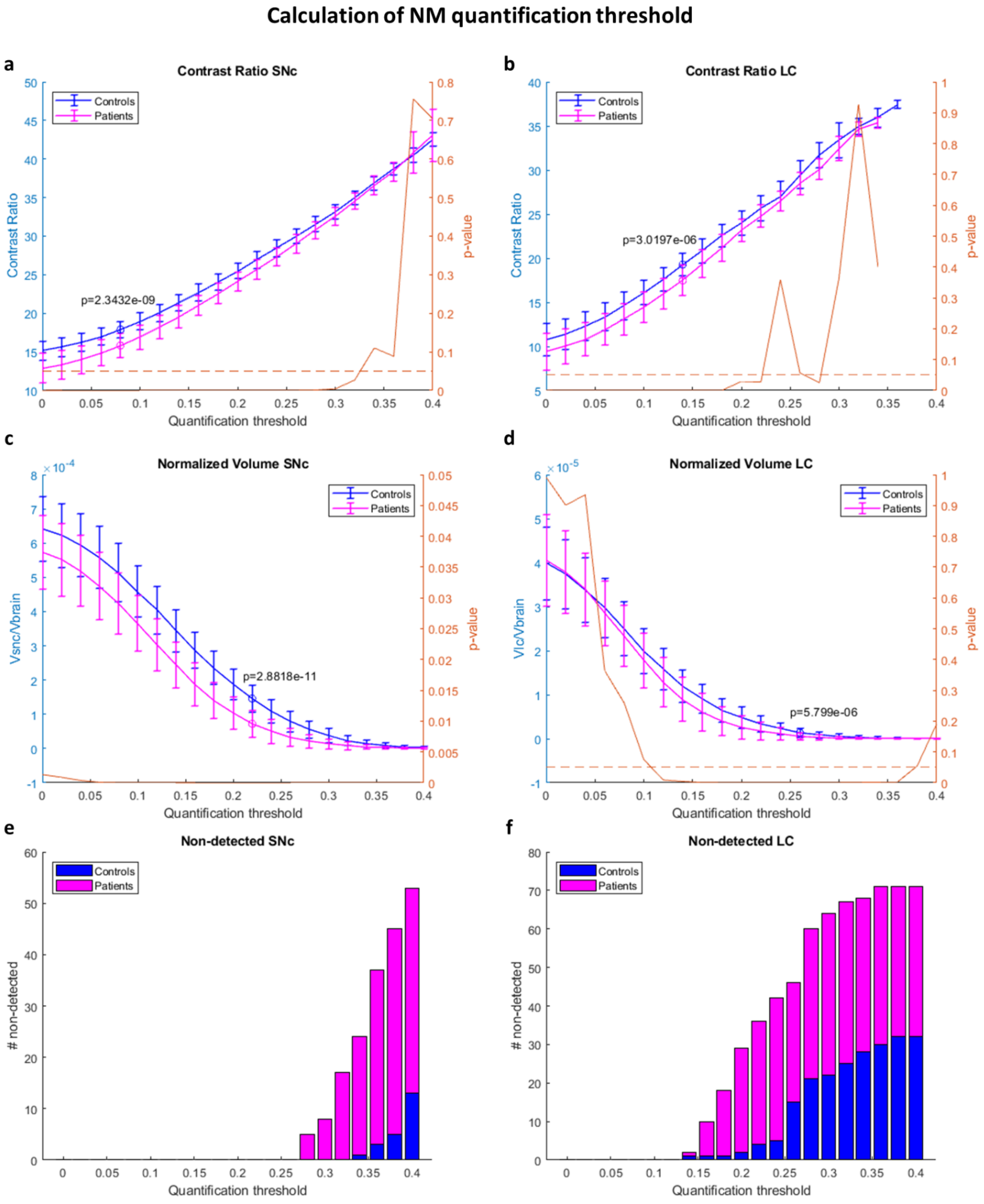

Supplementary Fig. 9. Optimal threshold for NM quantification in SNc and LC, based on its ability to discriminate HC from PD in terms of volume and contrast ratio variables. The threshold allows quantifying only hyperintense voxels in the segmented structure (i.e.,

those brighter than the average brainstem intensity by the ratio defined by the threshold). Parameter curves in reference to increasing thresholds: **a** SNc contrast ratio; **b** LC contrast ratio; **c** SNc normalized volume; **d** LC normalized volume; **e** Number of images for which no hyperintense voxels are detected in the SNc; and **f** Number of images for which no hyperintense voxels are detected in the LC. HC and PD curves are shown in blue and red, respectively. P-value curves of the Wilcoxon rank sum test comparing HC and PD for each threshold are shown in orange, with their corresponding p-value axis on the right and the p=0.05 significance threshold as a dotted horizontal line. For each curve (**a-d**), the optimal threshold corresponding to the lowest p-value is marked with an empty circle. Note that a single optimal threshold is selected for NM quantification based on all the curves; in this case, 0.14 is chosen as the optimal NM quantification threshold.

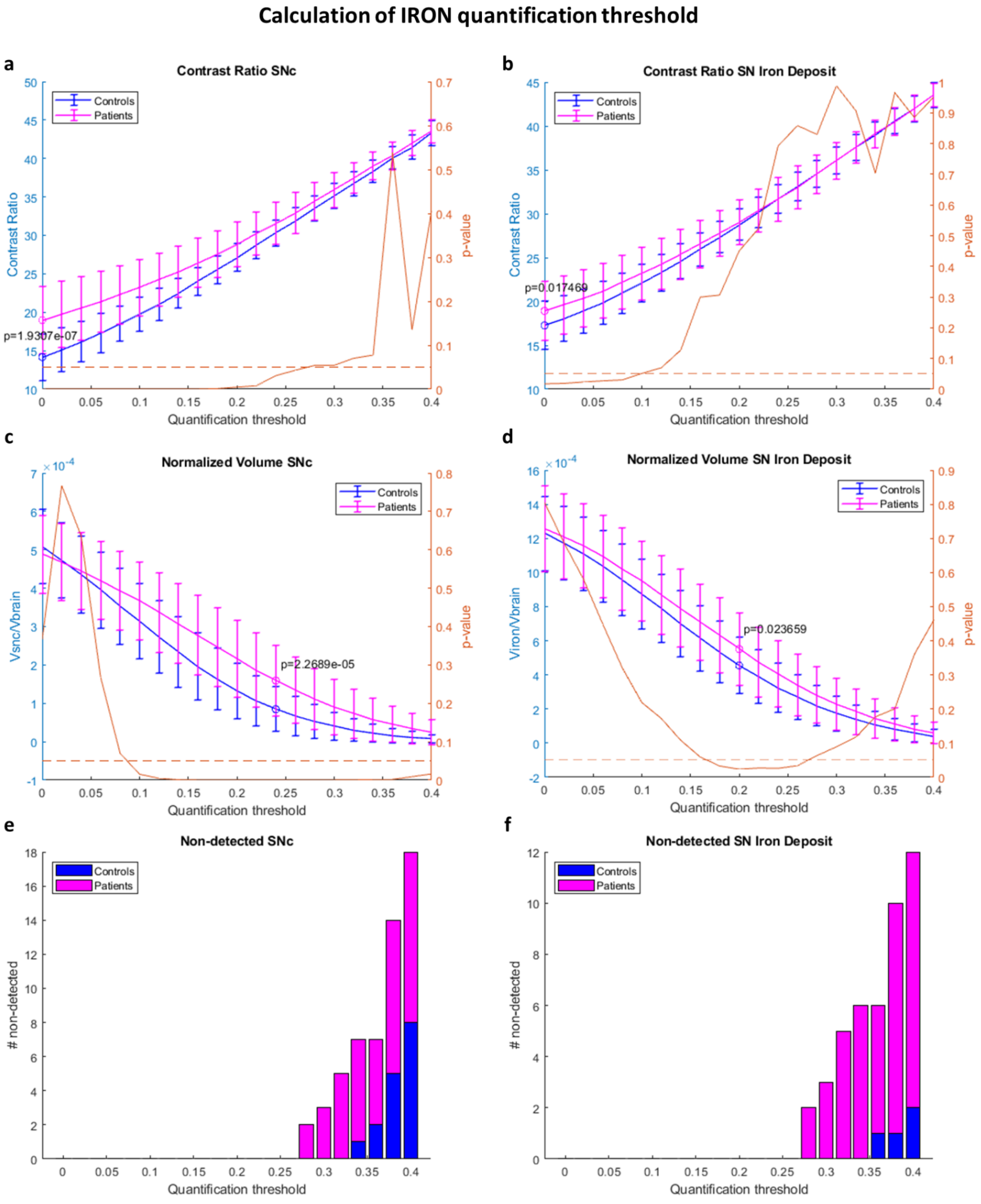

Supplementary Fig. 10. Calculation of the optimal threshold for iron quantification in SNc and whole SN iron deposit, based on the ability to discriminate HC and PD in terms of volume and contrast ratio. The threshold allows quantifying only hypointense voxels in the

segmented structure (i.e., those darker than the average brainstem intensity by the ratio defined by the threshold). Parameter curves with respect to increasing thresholds are represented: **a** Contrast ratio of the SNc; **b** Contrast ratio of the whole iron deposit in SN; **c** Normalised volume of the SNc; **d** Normalised volume of the whole iron deposit in SN; **e** Number of images for which no hypointense voxels are detected in the SNc; and **f** Number of images for which no hypointense voxels are detected in the whole iron deposit in SN. HC and PD curves are shown in blue and red, respectively. P-value curves of the Wilcoxon rank sum test comparing HC and PD for each threshold are shown in orange, with their corresponding p-value axis on the right and the p=0.05 significance threshold as a dotted horizontal line. For each curve (**a**-**d**), the optimal threshold corresponding to the lowest p-value is marked with an empty circle. Note that a single optimal threshold is selected for iron quantification based on all the curves; in this case, 0.12 is chosen as the optimal iron quantification threshold.

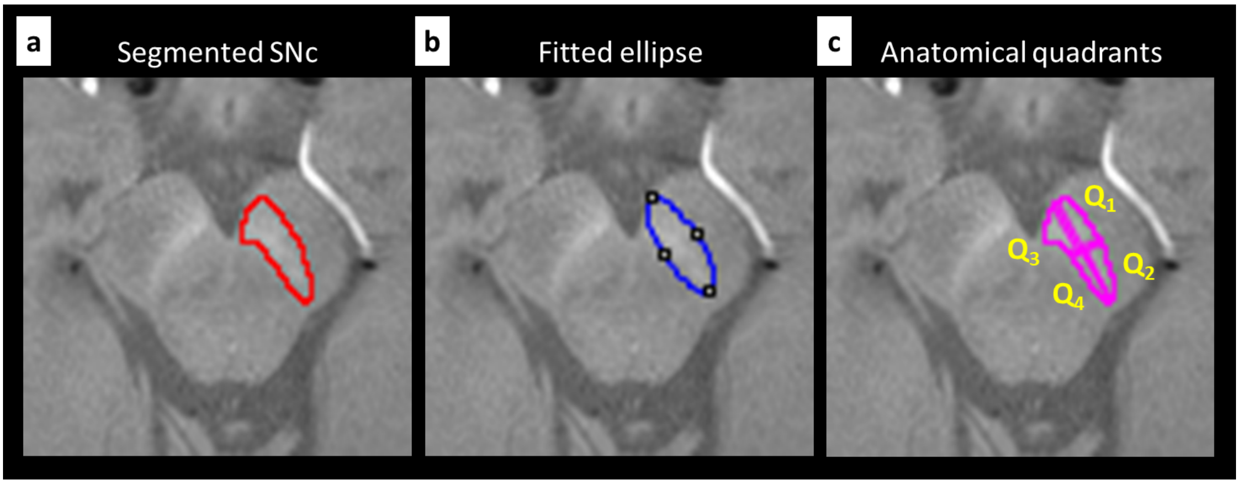

Supplementary Fig. 11. Elliptical-section cylinder fitting to delimit the four anatomical quadrants, for the analysis of the spatial distribution of NM and iron in the SNc. **a** Automatic segmentation of the right SNc; **b** Ellipse fitted to the segmented contour of the SNc; **c** Delimitation of the four anatomical quadrants of the SNc based on the major and minor axes of the fitted ellipse. Note that the example shows one 2D-slice for simplicity, but a 3D elliptical-section cylinder is actually fitted to the whole SNc, and thus the intersection between major and minor axes is common to all slices.

Supplementary Table 1. Median and interquartile range (IQR) group values of the CR and nVol of brainstem structures, with group comparisons by means of robust ANOVAs and post-hocs by means of independent robust t-tests.

Sequence	Region	Parameter	HC	iPD	LRRK2-PD	Group comparison	Post-hocs
NM	SNc	CR	21.26 (1.63)	19.47 (1.58)	19.50 (2.17)	F=28.72, p<0.001, ξ=0.67, Bootstrap CI = [0.47-0.86]	HC > iPD (p<0.001, d=0.76)<br>HC > LRRK2-PD (p<0.001, d=0.75)
		nVol	$3.44x10^{-4}$ ($9.68x10^{-5}$)	$2.57x10^{-4}$ ($1.04x10^{-4}$)	$2.39x10^{-4}$ ($8.46x10^{-5}$)	F=19.66, p<0.001, ξ=0.74, Bootstrap CI = [0.56 - 0.87]	HC > iPD (p<0.001, d=0.71)<br>HC > LRRK2-PD (p<0.001, d=0.93)
	LC	CR	19.09 (2.15)	17.36 (2.04)	18.60 (2.72)	F=14.14, p<0.001, ξ=0.54, Bootstrap CI = [0.34-0.72]	HC > iPD (p<0.001, d=0.72)<br>LRRK2-PD > iPD (p=0.04, d=0.42)
		nVol	$1.21x10^{-5}$ ($3.42x10^{-6}$)	$7.42x10^{-6}$ ($6.80x10^{-6}$)	$1.17x10^{-5}$ ($7.34x10^{-6}$)	F=7.65, p=0.002, ξ=0.48, Bootstrap CI = [0.16-0.68]	HC > iPD (p=0.002, d=0.63)<br>LRRK2-PD > iPD (p=0.03, d=-0.47)
Iron	SNc	CR	20.75 (2.81)	23.28 (5.10)	23.67 (4.46)	F=11.37, p<0.001, ξ=0.55, Bootstrap CI = [0.380.68]	iPD > HC (p=0.002, d=0.55)<br>LRRK2-PD > HC (p=0.002, d=0.75)
		nVol	$2.72x10^{-4}$ ($9.63x10^{-5}$)	$3.09x10^{-4}$ ($1.96x10^{-4}$)	$3.42x10^{-4}$ ($8.06x10^{-5}$)	F=9.21, p<0.001, ξ=0.42, Bootstrap CI = [0.21-0.66]	iPD > HC (p=0.03, d=0.39)<br>LRRK2-PD > HC (p<0.001, d=0.64)
	RN	CR	20.5 (2.97)	19.35 (3.65)	21.81 (3.89)	F=3.39, p= 0.045, ξ=0.40,	LRRK2-PD > iPD (p=0.047, d=0.46)

				Bootstrap CI = [0.09-0.66]	
nVol	$2.91x10^{-4}$ ($1.30x10^{-4}$)	$2.72x10^{-4}$ ($1.27x10^{-4}$)	$3.37x10^{-4}$ ($1.69x10^{-4}$)	F=2.51, p= 0.097 ξ=0.3, Bootstrap CI = [0.08-0.52]	-

Supplementary Table 2. Two-way (group x side) mixed ANOVAs of brainstem parameters, with main and interaction effects.

Sequence	Region	Parameter	Main effect of group	Main effect of side	Interaction group x side
NM	SNc	CR	*F=27.94, p<0.001*	*F=0.31, p=0.581*	*F=2.90, p=0.067*
		nVol	*F=20.47, p<0.001*	*F=33.01., p<0.001*	*F=4.77, p=0.014*
	LC	CR	*F=11.63, p<0.001*	*F=1.66, p=0.204*	*F=0.04, p=0.956*
		nVol	*F=9.62, p<0.001*	*F=135.19, p<0.001*	*F=0.24, p=0.784*
Iron	SNc	CR	*F=11.44, p<0.001*	*F=0.11, p=0.737*	*F=1.37, p=0.265*
		nVol	*F=8.69, p<0.001*	*F=61.87, p<0.001*	*F=0.08, p=0.922*
	RN	CR	*F=3.76, p=0.032*	*F=0.39, p=0.535*	*F=0.42, p=0.661*
		nVol	*F=2.80, p=0.07*	*F=0.10, p=0.748*	*F=0.91, p=0.410*

Supplementary Table 3. Two-way (group x sequence) mixed ANOVAs of the SNc CR and nVol, with main and interaction effects.

**Brainstem ROI**	**Parameter**	**Main effect of group**	**Main effect of sequence**	**Interaction group x sequence**
SNc	CR	*F=1.19, p=0.314*	*F=45.35, p<0.001*	*F=25.73, p<0.001*
	nVol	*F=0.53, p=0.595*	*F=7.87, p=0.007*	*F=37.85, p<0.001*

Supplementary Table 4. Results of ROC analyses performed, on individual NM or iron quantified parameters, and on the combination of parameters in different models through binary logistic regression, to examine the discriminatory power of 3D-ABSP for HC, iPD, and LRRK2-PD pairwise comparisons, and for HC vs. the whole PD patient group. Note that LogReg means binary logistic regression model combining the parameters between []. Note also that, in these LogReg models, notation of CR and nVol has been omitted for clarity (e.g., LogReg [SNc NM] means that SNc NM CR and SNc NM nVol have been combined in a binary logistic regression model.

**Group Comparison**	**Parameter or Regression Model**	**AUC**	**Confidence Interval**	**p-value**
HC vs. iPD	LC NM CR	0.835	[0.735-0.917]	<0.001
	LC NM nVol	0.744	[0.629-0.846]	<0.001
	LogReg [LC NM]	0.829	[0.696-0.903]	<0.001
	LogReg [LC NM + RN iron]	0.830	[0.720-0.916]	<0.001
	LogReg [LC NM + RN iron + SNc NM]	0.928	[0.826-0.974]	<0.001
	LogReg [LC NM + RN iron + SNc NM + SNc iron]	0.928	[0.833-0.975]	<0.001
HC vs. LRRK2-PD	LC NM CR	0.645	[0.473-0.780]	0.066

	LC NM nVol	0.512	[0.345-0.672]	0.896
	LogReg [LC NM]	0.695	[0.527-0.818]	0.020
	LogReg [LC NM + RN iron]	0.734	[0.578-0.847]	0.002
	LogReg [LC NM + RN iron + SNc NM]	0.930	[0.830-0.974]	<0.001
	LogReg [LC NM + RN iron + SNc NM + SNc iron]	0.970	[0.909-0.992]	<0.001
iPD vs. LRRK2-PD	LC NM CR	0.683	[0.509-0.793]	0.010
	LC NM nVol	0.705	[0.565-0.828]	0.008
	LogReg [LC NM]	0.703	[0.561-0.819]	0.008
	LogReg [LC NM + RN iron]	0.770	[0.624-0.879]	<0.001
	LogReg [LC NM + RN iron + SNc NM]	0.765	[0.597-0.871]	<0.001
	LogReg [LC NM + RN iron + SNc NM + SNc iron]	0.785	[0.653-0.879]	<0.001
HC vs. PD	SNc NM CR	0.856	[0.763-0.925]	<0.001
	SNc NM nVol	0.860	[0.769-0.920]	<0.001

	SNc iron CR	0.767	[0.655-0.854]	<0.001
	SNc iron nVol	0.688	[0.559-0.788]	<0.001
	LC NM CR	0.762	[0.655-0.845]	<0.001
	LC NM nVol	0.655	[0.507-0.756]	0.010
	RN iron CR	0.533	[0.412-0.658]	0.558
	RN iron nVol	0.565	[0.453-0.700]	0.286
	LogReg [SNc NM]	0.887	[0.809-0.941]	<0.001
	LogReg [SNc iron]	0.767	[0.651-0.851]	<0.001
	LogReg [LCNM]	0.766	[0.659-0.846]	<0.001
	LogReg [SNc NM + SNc iron]	0.926	[0.858-0.968]	<0.001
	LogReg [SNc NM + SNc iron + LC NM]	0.934	[0.851-0.971]	<0.001
	LogReg [SNc NM + SNc iron + LC NM + RN iron]	0.935	[0.873-0.971]	<0.001

Supplementary Table 5. Multiple regression analyses of the brainstem MRI measures (explanatory variables). The second column shows the statistics of the regression model when all predictors were included. The third column specifies the variables suggested by the feature selection algorithm. The fourth column shows the statistics of the regression model when only the selected variables were included. The last column shows the statistics of the significant predictors on each explanatory variable. F: F-statistic, p: p-value, R^2: adjusted R-Squared; β: standardized coefficients; b: non-standardized coefficients.

**Explanatory variable**	**Model statistics (all predictors)**	**Feature selection meaningful variables**	**Model statistics (selected variables)**	**Significant predictors**
SNc NM CR	$F_{20,42}=1.58$, $p=0.105$, $R^2=0.158$	Psychiatric symptoms	$F_{1,61}=7.20$, $p=0.009$, $R^2=0.091$	Psychiatric symptoms (b = -1.63, p=0.009)
SNc NM nVol	$F_{20,42}=0.59$, $p=0.90$, $R^2=-0.153$	-	-	-
SNc iron CR	$F_{20,42}=1.46$, $p=0.151$, $R^2=0.128$	Age, disease duration, psychiatric symptoms	$F_{3,59}=3.31$, $p=0.026$, $R^2=0.10$	Disease duration (β = 0.42, p=0.012)
SNc iron nVol	$F_{20,42}=2.27$, $p=0.012$, $R^2=0.291$	Age, disease duration, psychiatric symptoms, PD-CRS	$F_{4,58}=7.16$, $p<0.001$, $R^2=0.284$	Age (β = -0.42, p<0.001)<br>Disease duration (β = 0.41, p<0.001)

LC NM CR	$F_{20,42}$=1.55, p=0.114, R^2=0.151	-	-	-
LC NM nVol	$F_{20,42}$=1.62, p=0.093, R^2=0.167	Hiposmia, sex	$F_{3,59}$=3.63, p=0.032, R^2=0.078	Sex (b = -2.5e-6, p=0.041)
RN iron CR	$F_{20,42}$=1.72, p=0.07, R^2=0.188	-	-	-
RN iron nVol	$F_{20,42}$=2.02, p=0.027, R^2=0.248	PD-CRS, MMSE, age	$F_{3,59}$=4.81, p=0.005, R^2=0.156	-
