# Supplementary material for: Brainstem neuromelanin and iron MRI reveals a precise signature for idiopathic and LRRK2 Parkinson’s disease
Source: NPJ Parkinsons Dis. 2023 Apr 15;9:62. doi: 10.1038/s41531-023-00503-2 (PMC10105708; doi:10.1038/s41531-023-00503-2)
Supplement: Supplementary file 2 — Consortium description [file 41531_2023_503_MOESM2_ESM.docx]

**Apendix 1**

**Catalonian Neuroimaging Parkinson’s disease Consortium**

| **Name** | **E-Mail** | **Filiation** |
| --- | --- | --- |
| Berta Pascual-Sedano | **BPascual@santpau.cat** | Movement Disorders Unit, Neurology, Hospital Santa Creu i Sant Pau, Universitat Autònoma de Barcelona, Barcelona, Catalonia, Spain. |
| Juan Marin | **jmarinl@santpau.cat** | Movement Disorders Unit, Neurology, Hospital Santa Creu i Sant Pau, Universitat Autònoma de Barcelona, Barcelona, Catalonia, Spain. |
| Asuncion Avila | **asuncion.avila@sanitatintegral.org** | Neurology, Complex Hospitalari Moisès Broggi, Sant Joan Despí, Barcelona, Spain |
| Mariateresa Buongiorno | **mtbuongiorno@mutuaterrassa.cat** | Movement Disorders Unit, Neurology, Hospital Universitari Mútua de Terrassa, Terrassa, Barcelona, Spain |
| Juan Pablo Tartari | **juanpablo.tartari@gmail.com** | Movement Disorders Unit, Neurology, Hospital Universitari Mútua de Terrassa, Terrassa, Barcelona, Spain |
| Victor Puente | **Vpuente@parcdesalutmar.cat** | Movement Disorders Unit, Neurology, Hospital del Mar, Barcelona, Spain. |
| Mario Ezquerra | **EZQUERRA@clinic.cat** | Parkinson's Disease and Movement Disorders Unit, Neurology, Hospital Clínic de Barcelona, Barcelona, Catalonia, Spain |
| Francesc Valldeoriola | **FVALLDE@clinic.cat** | Parkinson's Disease and Movement Disorders Unit, Neurology, Hospital Clínic de Barcelona, Barcelona, Catalonia, Spain |
| Yaroslau Compta | **YCOMPTA@clinic.cat** | Parkinson's Disease and Movement Disorders Unit, Neurology, Hospital Clínic de Barcelona, Barcelona, Catalonia, Spain |
| Eduard Tolosa | **ETOLOSA@clinic.cat** | Parkinson's Disease and Movement Disorders Unit, Neurology, Hospital Clínic de Barcelona, Barcelona, Catalonia, Spain |
| Claustre Pont | **mcpont@fphag.org** | Neurology Service, Hospital General de Granollers, Granollers, Barcelona, Spain). |
