# Supplementary material for: Brainstem neuromelanin and iron MRI reveals a precise signature for idiopathic and LRRK2 Parkinson’s disease
Source: NPJ Parkinsons Dis. 2023 Apr 15;9:62. doi: 10.1038/s41531-023-00503-2 (PMC10105708; doi:10.1038/s41531-023-00503-2)
Supplement: Supplementary file 3 — Reporting Summary [file 41531_2023_503_MOESM3_ESM.pdf]

nature portfolio

Corresponding author(s): Maria A. Pastor

Last updated by author(s): Jul 27, 2020

Reporting Summary

Nature Portfolio wishes to improve the reproducibility of the work that we publish. This form provides structure for consistency and transparency in reporting. For further information on Nature Portfolio policies, see our Editorial Policies and the Editorial Policy Checklist.

Statistics

For all statistical analyses, confirm that the following items are present in the figure legend, table legend, main text, or Methods section.

n/a	Confirmed	
☐	☒	The exact sample size (*n*) for each experimental group/condition, given as a discrete number and unit of measurement
☐	☒	A statement on whether measurements were taken from distinct samples or whether the same sample was measured repeatedly
☐	☒	The statistical test(s) used AND whether they are one- or two-sided<br>*Only common tests should be described solely by name; describe more complex techniques in the Methods section.*
☐	☒	A description of all covariates tested
☐	☒	A description of any assumptions or corrections, such as tests of normality and adjustment for multiple comparisons
☐	☒	A full description of the statistical parameters including central tendency (e.g. means) or other basic estimates (e.g. regression coefficient) AND variation (e.g. standard deviation) or associated estimates of uncertainty (e.g. confidence intervals)
☐	☒	For null hypothesis testing, the test statistic (e.g. *F*, *t*, *r*) with confidence intervals, effect sizes, degrees of freedom and *P* value noted<br>*Give P values as exact values whenever suitable.*
☒	☐	For Bayesian analysis, information on the choice of priors and Markov chain Monte Carlo settings
☐	☒	For hierarchical and complex designs, identification of the appropriate level for tests and full reporting of outcomes
☒	☐	Estimates of effect sizes (e.g. Cohen's *d*, Pearson's *r*), indicating how they were calculated

Our web collection on statistics for biologists contains articles on many of the points above.

Software and code

Policy information about availability of computer code

Data collection	No software was used for data collection.
Data analysis	Data analysis was performed in R (4.0.1) and in Matlab (R2021a).

For manuscripts utilizing custom algorithms or software that are central to the research but not yet described in published literature, software must be made available to editors and reviewers. We strongly encourage code deposition in a community repository (e.g. GitHub). See the Nature Portfolio guidelines for submitting code & software for further information.

Data

Policy information about availability of data

All manuscripts must include a data availability statement. This statement should provide the following information, where applicable:

- Accession codes, unique identifiers, or web links for publicly available datasets
- A description of any restrictions on data availability
- For clinical datasets or third party data, please ensure that the statement adheres to our policy

Written requests for access to the data reported in this paper will be considered upon request to the corresponding author MAP, COS and PP as long as the appropriateness of the use of data exists. If the use is appropriate, a data-sharing agreement will be signed before a fully de-identified version of the dataset used for analysis is available.

Human research participants

Policy information about studies involving human research participants and Sex and Gender in Research.

Reporting on sex and gender	In the present manuscript, we only use the term "sex" (biological attribute).
Population characteristics	We used the covariates of age, and the genotypic information for patients and healthy controls. We used also past and current diagnosis of Parkinson disease for the patients.
Recruitment	Subjects with Parkinson disease with or without mutations in LRRK2 or GBA. Their healthy spouses were invited to participate in the study. Bias selection was performed in order to match the groups by age and sex.
Ethics oversight	The study was approved by the University of Navarra Research Ethics Committee

Note that full information on the approval of the study protocol must also be provided in the manuscript.

Field-specific reporting

Please select the one below that is the best fit for your research. If you are not sure, read the appropriate sections before making your selection.

☒ Life sciences ☐ Behavioural & social sciences ☐ Ecological, evolutionary & environmental sciences

For a reference copy of the document with all sections, see nature.com/documents/nr-reporting-summary-flat.pdf

Life sciences study design

All studies must disclose on these points even when the disclosure is negative.

Sample size	We calculated the optimal sample size of the corresponding expected effect size with G*Power 3.1.9.6 software based in our previous study.
Data exclusions	No data were excluded from the analysis.
Replication	We stated in the manuscript that findings need to be replicated.
Randomization	Allocation was not random. We controlled for age and sex between HC and PD groups.
Blinding	The investigators were blinded to group allocation during data collection and analysis.

Reporting for specific materials, systems and methods

We require information from authors about some types of materials, experimental systems and methods used in many studies. Here, indicate whether each material, system or method listed is relevant to your study. If you are not sure if a list item applies to your research, read the appropriate section before selecting a response.

Materials & experimental systems

n/a	Involved in the study	
☒	☐	Antibodies
☒	☐	Eukaryotic cell lines
☒	☐	Palaeontology and archaeology
☒	☐	Animals and other organisms
☐	☒	Clinical data
☒	☐	Dual use research of concern

Methods

n/a	Involved in the study	
☒	☐	ChIP-seq
☒	☐	Flow cytometry
☐	☒	MRI-based neuroimaging

Clinical data

Policy information about clinical studies

All manuscripts should comply with the ICMJE guidelines for publication of clinical research and a completed CONSORT checklist must be included with all submissions.

Clinical trial registration	Not applicable
Study protocol	*Note where the full trial protocol can be accessed OR if not available, explain why.*

Data collection	*Describe the settings and locales of data collection, noting the time periods of recruitment and data collection.*
Outcomes	*Describe how you pre-defined primary and secondary outcome measures and how you assessed these measures.*

Magnetic resonance imaging

Experimental design

Design type	*Indicate task or resting state; event-related or block design.*
Design specifications	*Specify the number of blocks, trials or experimental units per session and/or subject, and specify the length of each trial or block (if trials are blocked) and interval between trials.*
Behavioral performance measures	*State number and/or type of variables recorded (e.g. correct button press, response time) and what statistics were used to establish that the subjects were performing the task as expected (e.g. mean, range, and/or standard deviation across subjects).*

Acquisition

Imaging type(s)	Structural
Field strength	3T
Sequence & imaging parameters	MPRAGE (T1 sequence). 3D-NM sensitive T1-weighted turbo spin-echo sequence (NM-sequence), and long-TE high-resolution fully flow-compensated three-dimensional (3D) GRE (SWI sequence). See supplementary information.
Area of acquisition	Whole brain scan (without cerebellum). Acquisition perpendicular from fourth ventricle.

Diffusion MRI ☐ Used ☒ Not used

Preprocessing

Preprocessing software	SPM12 and Matlab R2021a.
Normalization	Individual normalization of the ROIs volume to intracraneal brain volume.
Normalization template	Not applicable.
Noise and artifact removal	Realignment and sequence averaging.
Volume censoring	*Define your software and/or method and criteria for volume censoring, and state the extent of such censoring.*

Statistical modeling & inference

Model type and settings	Multivariate.
Effect(s) tested	ANOVA and logistic regression on MRI parameters (CR and nVol).

Specify type of analysis: ☐ Whole brain ☒ ROI-based ☐ Both

Anatomical location(s): Substantia nigra, locus coeruleus, red nucleus.

Statistic type for inference (See Eklund et al. 2016)	Voxel-wise.
Correction	In robust analyses, confidence intervals and p-values were adjusted for multiple testing; also Bootstraping was used.

Models & analysis

n/a	Involved in the study
☒	☐ Functional and/or effective connectivity
☒	☐ Graph analysis
☐	☒ Multivariate modeling or predictive analysis

Multivariate modeling and predictive analysis	Demographic and clinical variables were used as independent variables in multiple regression analyses, whereas feature extraction was performed using Boruta's algorithm.
